# Evolution of Associative Learning in Chemical Networks

**DOI:** 10.1371/journal.pcbi.1002739

**Published:** 2012-11-01

**Authors:** Simon McGregor, Vera Vasas, Phil Husbands, Chrisantha Fernando

**Affiliations:** 1 Department of Informatics, University of Sussex, Falmer, Brighton, United Kingdom; 2 Departament de Genètica i de Microbiologia, Grup de Biologia Evolutiva (GBE), Universitat Autònoma de Barcelona, Bellaterra, Barcelona, Spain; 3 School of Electronic Engineering and Computer Science (EECS), Queen Mary, University of London, London, United Kingdom; Research Center Jülich, Germany

## Abstract

Organisms that can learn about their environment and modify their behaviour appropriately during their lifetime are more likely to survive and reproduce than organisms that do not. While associative learning – the ability to detect correlated features of the environment – has been studied extensively in nervous systems, where the underlying mechanisms are reasonably well understood, mechanisms within single cells that could allow associative learning have received little attention. Here, using *in silico* evolution of chemical networks, we show that there exists a diversity of remarkably simple and plausible chemical solutions to the associative learning problem, the simplest of which uses only one core chemical reaction. We then asked to what extent a linear combination of chemical concentrations in the network could approximate the ideal Bayesian posterior of an environment given the stimulus history so far? This Bayesian analysis revealed the ‘memory traces’ of the chemical network. The implication of this paper is that there is little reason to believe that a lack of suitable phenotypic variation would prevent associative learning from evolving in cell signalling, metabolic, gene regulatory, or a mixture of these networks in cells.

## Author Summary

Whilst one may have believed that associative learning requires a nervous system, this paper shows that chemical networks can be evolved *in silico* to undertake a range of associative learning tasks with only a small number of reactions. The mechanisms are surprisingly simple. The networks can be analysed using Bayesian methods to identify the components of the network responsible for learning. The networks evolved were simpler in some ways to hand-designed synthetic biology networks for associative learning. The motifs may be looked for in biochemical networks and the hypothesis that they undertake associative learning, e.g. in single cells or during development may be legitimately entertained.

## Introduction

Here we evolve chemical networks in simulation to undertake associative learning. We define learning as the process by which information about the world is encoded into internal state (a memory-trace) in order to behave more adaptively in the future. Associative learning is learning of a relation between two types of event. Remarkably, the most frequently found circuits consisted of only one or two core chemical reactions responsible for learning, the other reactions being involved in subsidiary functions such as signal transduction. This is functionally simpler than the previously hand-designed biochemical circuits for classical conditioning that require several chemical reactions to implement Hebbian learning (a term which we use to refer to a mechanism that ensures that event A co-occurring with event B results in a greater probability that event B will occur given future presentations of A alone [1], [2]). Thus, this is a beautiful example of how evolution can find elegant solutions.

Chemical kinetics is Turing complete and therefore any computable mechanism for associative learning is theoretically possible [3], [4], however, this says nothing about which kinds of chemical mechanisms for learning are likely to evolve. Here we use *in silico* natural selection [5], [6], [7], [8], [9] to evolve chemical networks that are selected on the basis of their ability to carry out various associative learning tasks. Also known as genetic algorithms [10] or evolutionary computation [11], [12], the principle follows that of selective breeding. An initial random population of chemical networks is constructed. Each network is assessed for its quality as defined by a ‘fitness function’ that maps quality to fitness. The next generation is produced by allowing networks to replicate with mutation (and crossover) in proportion to their fitness. This process iterates for many generations, eventually producing higher quality networks that are capable of solving the desired task. The closest work to ours is the evolution of associative learning in continuous recurrent neural networks [13].

Our simulation evolves an abstract chemistry; however unlike many experiments with purely artificial chemistries [14], [15] it was designed to respect conservation of mass and energy, an essential consideration for transferring the insights from *in silico* models to chemical reality [16], [17], [18], which is our ultimate goal. Each ‘molecule’ consists of ‘0’ and ‘1’ atoms, and only the number of digits (and not their sequence) determines the species' identity. Any interchange of building blocks between molecules was allowed to happen in reactions. With the exception of the implicit decay reactions, all the simulated chemical reactions are reversible., However, some reactions may be effectively irreversible because the reaction rate in the backward direction is very low compared to the reaction rate in the forward direction. For details of the artificial chemistry model refer to the Methods section. Results from a pilot study with simpler chemistry are described in Supporting Information TextS1, part 1.

Traditionally there are two types of associative learning, classical and instrumental conditioning, the former involves passive observation of events, e.g. associating the sound of a bell with the smell of food, and the later involves relating self-generated actions and their consequences, e.g. learning that pressing a lever produces food [19]. We developed tasks that evoke the classical conditioning paradigm in psychology [20]. The network receives input from the environment (in the form of chemical boluses externally introduced into the system) and produces output (defined as the concentration of a particular chemical species measured over a particular test-period). The chemical dynamics of the system (the changes in concentration of chemical species) describe the behaviour of the network according to its sensory input.

In all the learning tasks, the chemical network had to learn to anticipate the injection of a control chemical C, known as the unconditioned stimulus UCS in the classical conditioning literature. Anticipation of C means to act in a manner that shows knowledge that certain events can predict C. Anticipation can be learned or innate. In our tasks it is necessary to learn to anticipate, not just to evolve innate temporal expectations. All tasks involve two possible conditions. In one condition the network should be able to use another chemical S (stimulus pulse), i.e. the conditioned stimulus CS, that reliably precedes C to predict the occurrence of C. Prediction results in the production of an output chemical O - the conditioned response CR - immediately after S is presented but prior to C. If this condition has been properly inferred, output chemical O should then be reliably elicited by the stimulus pulse S alone, after pairing S with C. This describes the “associated” condition. In the other, “non-associated” condition, S cannot theoretically be used to predict C. We therefore no not wish to see a CR (i.e. no output O production) following S. Thus, in all cases the network's fitness depends on whether it has learned the association between S and C by requiring it to produce an output chemical after S only when it is reliably followed by C, but not otherwise. There is no explicit training and testing phase in our experiments. The network's task is to respond appropriately as quickly as possible.

Consider a possible real-world example of how such functionality may be adaptive. Imagine that C (UCS) is a toxin and that S (CS) is a chemical that in some environments (but not others) can predict that toxin. Imagine that a metabolically expensive anti-toxin O (CR) can be synthesised to neutralise the toxin C. Then it would be advantageous to use S to initiate the synthesis of anti-toxin O in lieu of C in the environments in which S was predictive, but not in those environments in which S was not predictive, where instead the no O should occur, i.e. no production of anti-toxin in response to S. All tasks pose variants of this fundamental problem. The fact the network may find itself in either environment within a lifetime means that it could not evolve the simple strategy of sensitization where it always produces output chemical O in response to S. We used five different tasks, designed to provide a systematically more challenging associative learning problem. A summary of the tasks, and the information required for achieving maximal fitness on them (i.e. the simplest discrimination that is sufficient for optimal performance), is given in Table 1. The first two tasks do not require detection of a temporal correlation between S and C, i.e. they can be solved without associative learning, i.e. by sensitization/habituation alone. They demonstrate that in restricted environments, information about associations between things can be equivalent to information about simpler (lower-order) environmental features, such as the frequency of individual event types. However, the later three tasks are designed such that they necessitate discriminations based on observation of associations, e.g. discriminating environments in which S and C are temporally correlated compared to environments in which they occur independently. Thus, the final three tasks are true associative learning tasks that cannot be solved without the capacity to observe associations and modify ones behaviour accordingly.

**Table 1:** Five learning tasks of differing complexity on which chemical networks were evolved.

Task	Typical Inputs by Environment Type		Network required to determine:
	Unassociated	Associated	
Clocked [Non-associative]	S pulses alone	S→C pulse pairs	Do C pulses occur?
Noisy Clocked [Non-associative]	S pulses alone	S→C pulse pairs	Do C pulses occur more often than S pulses alone?
Non-Clocked [True Associative]	S and C pulses with independent timing	S→C pulse pairs	Do C pulses tend to occur shortly after S pulses?
AB-BA [True Associative]	C→S pulse pairs	S→C pulse pairs	Do S→C pulse pairs tend to occur more often than C→S pulses, over an extended period?
2-bit Environment [True Associative]	C→C, C→S and S→S pulse pairs	S→C pulse pairs	Do more S→C pulse pairs occur than any other type of input event?

Classical conditioning involves a wide range of different training and testing regimes, e.g. Pavlovian conditioning [21], Blocking [22], Backwards Blocking [23], overshadowing [19], etc. Typically these paradigms show an unconditioned response to the control (UCS). Above we have used a set of training and testing regimes that do not explicitly require an unconditioned response (UCR) to the UCS (control) molecule alone. In other words, we have assumed that a straightforward chemical reaction exists, independent of the network modelled, that is capable of producing an UCR to the control molecule. An important aspect of classical conditioning is extinction, a reduction in the conditioned response CR when the conditioned stimulus CS (stimulus) is repeatedly presented in the absence of the unconditioned stimulus UCS (control). All the networks presented here show extinction, even though they were not explicitly evolved on an extinction paradigm, see Supporting Information TextS1 Part 2.

### Clocked task

**Figure 1:**
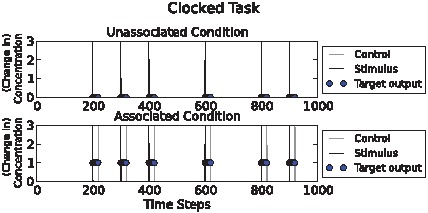
Clocked task illustration. Above: “unassociated” condition. Below: “associated” condition. Stimulus (“S”) (CS) and control (“C”) (UCS) pulses are shown as black and grey spikes respectively. Circles show (desired) target concentrations of the output chemical O (CR = high O). In the unassociatied condition, input S is given and the output chemical must remain low during the period when the output is assessed (blue circles). In the lower, associated condition, two inputs (S and C) are provided and the network must now produce a high output chemical concentration during the period the output is assessed (blue circles). Note that input S signals the onset of the period that the output chemical must have a high concentration, and input C signals the end of that period. The chemical network must use its knowledge of input C to determine its response to input S.

The times when the network must respond by producing an output O when stimulus S is associated with chemical C were constrained to regular “clock ticks” to make the task as easy as possible for the networks. Because there is no noise, this is a simple task as the very first input event (which is either S on its own, or S followed by C) provides all the necessary information for maximising fitness (Figure 1). The blue blobs show the time at which the target output is required, i.e. when the target output contributes to fitness. In the associated condition the target output is high (1) and in the unassociated condition the target output is low (0). At all other times it does not matter what the target output is. This was intended to give evolution more leeway by imposing fewer constraints. Even so, many evolved solutions maintained the output concentration at low levels when the target output was not evaluated.

### Noisy clocked task

**Figure 2:**
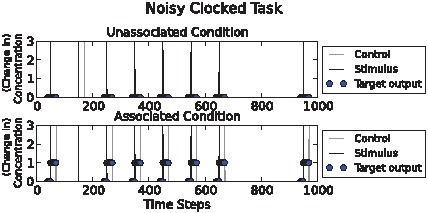
Noisy clocked task illustration. Above: “unassociated” condition. Below: “associated” condition. Stimulus (“S”) pulses and control (“C”) pulses are shown as black and grey spikes respectively. Circles show target output chemical concentration values. Note that the second input event, at time t = 150, (in both conditions) is a noisy event, either a false positive or a false negative control chemical pulse occurs. The environments can be distinguished on the fact that in the associated condition below the pulses co-occur with greater frequency than in the unassociated condition.

This task is identical to task 1., except that stimulus-control pulse pairs occurred with a low (non-zero) frequency in the unassociated environment and stimulus pulses without control pulses occurred with a low (non-zero) frequency in the associated environment. This produced ambiguity about the hidden state (which environment the network is in) on the basis of observed state variables (S and C pulses). Here, high fitness networks must consider more of the past, since isolated input events are unreliable indicators of the correct output chemical response (Figure 2.). A successful chemical network should update its ‘belief’ in which environment it is in on the basis of several observed associations, not just one; in other words, it must integrate information over time. For example, if we examine Figure 2., we see that the second stimulus pulse is followed by a control pulse even in the unassociated condition, and that the second stimulus pulse is not followed by a control pulse in the associated condition. However, the reader will notice that ‘cheating’ is possible in these two tasks because in the associated condition C occurs more often *in total* than in the unassociated condition, thus simply learning to respond to S when C is on average higher in concentration is a sufficient strategy. The temporal relation between S and C does not need to be learned here. This simple solution is excluded in the design of the next task.

### Non-clocked associative task

**Figure 3:**
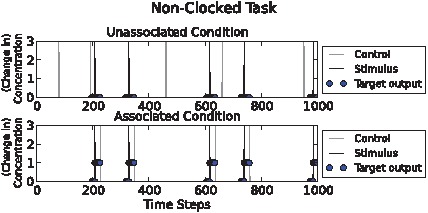
Non-clocked task illustration. Above: “Unassociated” condition. Below: “associated” condition. Stimulus (“S”) and control (“C”) pulses are shown as black and grey spikes respectively. Circles show target (desired) output chemical concentration values. Here, in the unassociated condition both C and S pulses occur, but C pulses do not reliably follow S pulses unlike the associated condition. Higher fitness could be achieved by detecting relational aspects of inputs, rather than simply observing the occurrence of control events as in the previous tasks.

In this task the timing of stimulus pulse and control pulse input events was unconstrained, and, most importantly, the unassociated and the associated environments received the same number of control pulses, except that in the unassociatied environment they were randomly distributed while in the associated environment they reliably followed stimulus pulses. Therefore this task was harder still, since it involved detecting relational aspects of inputs rather than merely first-order statistics of control pulses like the first two tasks (Figure 3).

### AB-BA task

**Figure 4:**
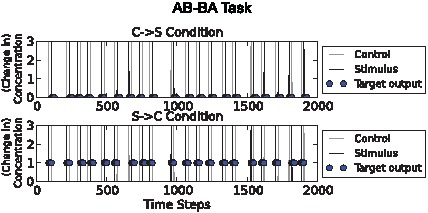
AB-BA task illustration. Above: “C→S” condition. Below: “S→C” condition. Stimulus (“S”) and control (“C”) pulses are shown as grey and black spikes respectively. Circles show target output values. This task spans a longer time period than the others, because it is noisier. In the C→S condition C pulses typically precede S pulses, whereas in the S→C condition, S pulses typically precede C pulses. The chemical network must produce a high output chemical concentration following the S pulse in the S→C condition but not in the C→S condition. The noise involves flipping of the order of S and C pulses so that S→C pulses sometimes occur in the “C→S” condition and vice versa. The noisiness can be controlled.

Like task 3., this task used unconstrained input timing with noise and required relations between inputs to be detected. The difference is that in the first environment, where the network was required to keep the output chemical concentration low, control pulses reliably preceded stimulus pulses (Figure 4) rather than the other way around. In both cases S and C are associated, but occur in a different temporal order. The network must distinguish between these two kinds of temporal relationship.

### “2-bit environment” task

**Figure 5:**
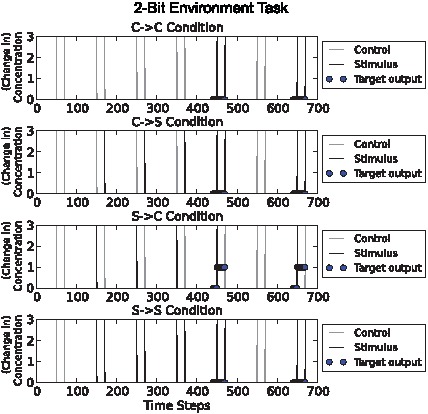
2-bit environment task illustration. Conditions from top to bottom: “C→C”, “C→S”, “S→C”, “S→S”. Stimulus (“S”) and control (“C”) pulses are shown as grey and black spikes respectively. Circles show target output values. In this task the only condition in which the output chemical should be high is where S pulses precede C pulses. Notice that we only assess the output during the second part of each condition, giving the network some time to make a judgement about which condition it is in. This task was designed by hand to provide a significant challenge while allowing exhaustive evaluation.

The previous tasks described classes of stochastically-generated environment. Hence, any one network could be evaluated only on a sample of the environments typical of the task. By contrast, this task was designed by hand to provide a significant challenge while allowing exhaustive evaluation. The networks performance was measured in four environments (all possible combinations of stimulus-control pulse pairs). Maximal fitness required accumulating relational data over multiple input events; the task was specifically designed to exclude strategies that rely on the first or most recent input event (Figure 5). Unlike the previous experiments, the network must learn 2 bits of information because it must distinguish one of 2^2^ states (not just 2^1^ states).

## Results

**Figure 6:**
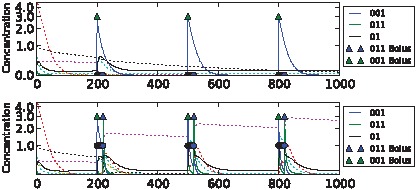
Sample dynamics of an evolved network for the clocked task. Upper: “unassociated” condition. Lower: “associated” condition. Black solid line shows output concentration; blue solid line shows stimulus concentration; green solid line shows control concentration. Dotted lines show intermediate chemical concentrations. Circles indicate target output values for the network. Triangles show input boluses.

**Figure 7:**
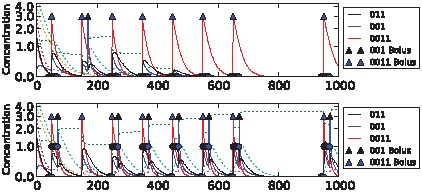
Sample dynamics of an evolved network for the noisy clocked task. Upper: “unassociated” condition. Lower: “associated” condition. Black solid line shows output concentration; red solid line shows stimulus concentration; blue solid line shows control concentration. Dotted lines show intermediate chemical concentrations. Circles indicate target output values for the network. Triangles show input boluses.

**Figure 8:**
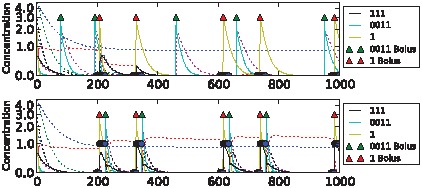
Sample dynamics of an evolved network for the non-clocked task. Upper: “unassociated” condition. Lower: “associated” condition. Black solid line shows output concentration; yellow solid line shows stimulus concentration; blue solid line shows control concentration. Dotted lines show intermediate chemical concentrations. Circles indicate target output values for the network. Triangles show input boluses.

**Figure 9:**
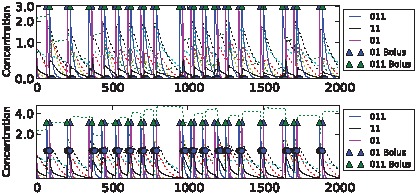
Sample dynamics of an evolved network for the AB-BA task. Upper: “unassociated” condition. Lower: “associated” condition. Black solid line shows output concentration; blue solid line shows stimulus concentration; purple solid line shows control concentration. Dotted lines show intermediate chemical concentrations. Circles indicate target output values for the network. Triangles show input boluses.

**Figure 10:**
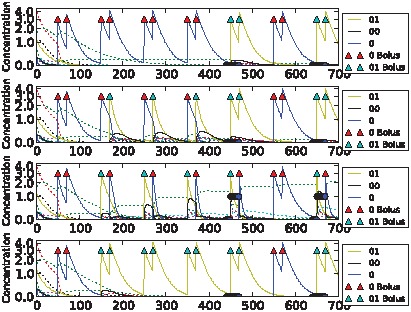
Sample dynamics of an evolved network for the 2-bit environment task. From top to bottom: C→C, C→S, S→C and S→S environments. Black solid line shows output concentration; yellow solid line shows stimulus concentration; blue solid line shows control concentration. Dotted lines show intermediate chemical concentrations. Circles indicate target output values for the network. Triangles show input boluses.

We were able to evolve highly fit networks for each of the tasks above. Dynamics of the best performing networks on the five different tasks are shown in Figures 6–10 (for details of the chemical networks see Supporting Information TextS1 Part 3). The networks display learning – changes in state which reflect the statistics of their past inputs, and determine their response to input boluses adaptively. Note that the network's performance typically increases over the evaluation period, suggesting that a long-term memory-trace builds up over consecutive stimulus-control pairs.

The differences in task difficulty can also be observed on the graphs. For the simplest, clocked, task one input event was enough for the network to decide about the environment; but for the AB-BA or the 2-bit task a much longer training period was required. Figure 6 shows the performance in the Clocked task. The output chemical O (molecule ‘01’) is shown in black. In the top (unassociated) condition after the first presentation of stimulus S and the absence of a control bolus, its concentration drops and never returns. In the associated environment below, the output chemical shows the opposite dynamics after the first paired input of S and C. Figure 7 shows the evolved performance of a network on the noisy clocked task. The output chemical is again shown in black, and again in the unassociated task its concentration gradually declines (except after a misleading S-C pair shown during the second input event). In the associated environment the black output chemical continues to be produced when the network is stimulated with S. Figure 8 shows performance on the non-clocked task where it is necessary to learn explicitly the temporal correlation between S and C because in both tasks the overall amount of S and C is the same. Again an evolved network is successful in this because the black output chemical is only produced in the associated condition below and not in the unassociated condition above. Figure 9 shows the performance of a network that successfully evolved to solve the AB-BA task. The concentration of the output chemical in the lower condition is higher on average than the output concentration in the upper condition. The performance was only assessed during the second half of the task and this is where the greatest difference in black chemical output is seen. Figure 10 shows successful performance on the 2-bit environment task with the black output chemical only showing high concentration in the third condition.

### Network structure

**Figure 11:**
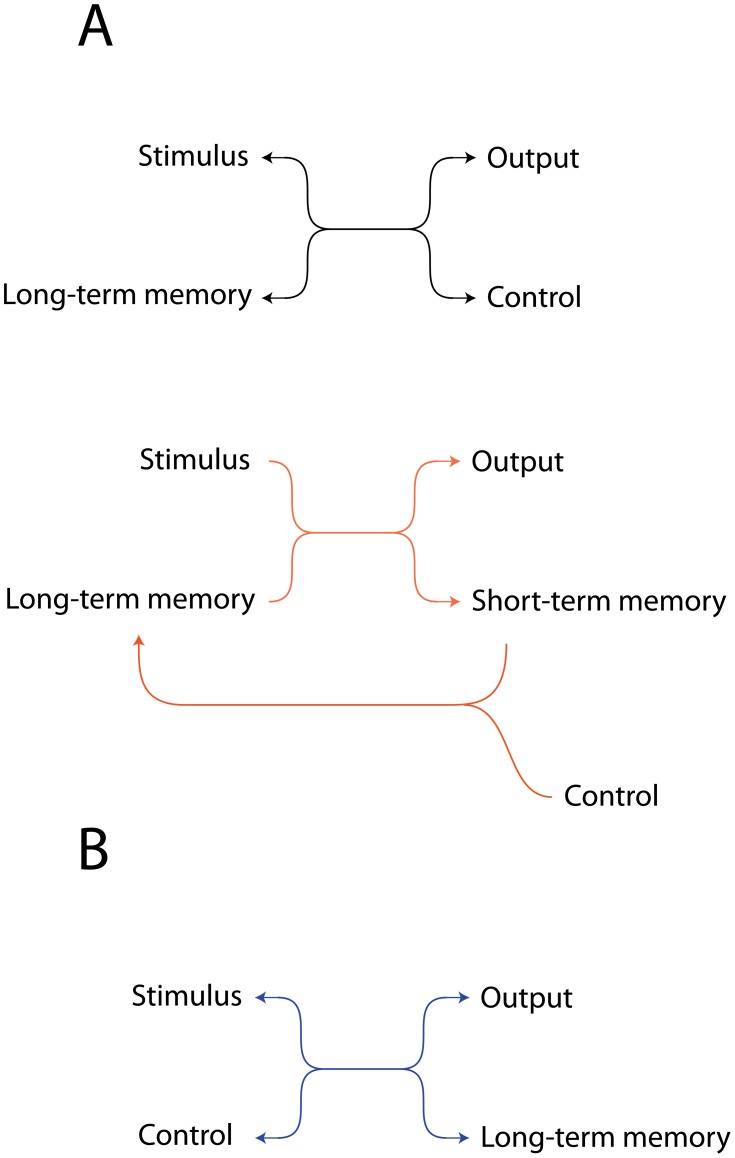
Chemical motifs for associative learning. (A) A long-term memory chemical could be identified in most networks: this reacted with the stimulus to produce output, and was generated only in the “associated” environment. *Top.* A simple reversible reaction in which stimulus+slow decaying memory-trace molecule produce output, and the control molecule regenerates the memory molecule for reuse. *Bottom.* Two *almost* irreversible reactions allow an improvement on the previous motif because here the decay rate of the output chemical is made independent of the decay rate of a short-term memory molecule, allowing decoupling of the control from the output molecule. (B) In several networks the overlap between the signal and control initialized long-term memory production. Again, a single reversible reaction but with S and C reacting together. It works because the control chemical decays quickly but the stimulus molecule decays slowly. Therefore stimulus and control molecules only co-occur when control follows stimulus, and not when stimulus follows control.

Having evolved approximately 10 networks capable of solving each task, we ask, how do they work? The evolutionary algorithm permitted increases or decreases in the number of chemical species and the number of chemical reactions, see Methods. The smallest evolved network required only two reactions, but the typical number of reactions in an evolved network was 12 (mean 11.9, median 12). A greedy pruning algorithm applied to the networks revealed that most of these reactions were superfluous; typically only 5 reactions (mean 4.7, median 5) were necessary to achieve a fitness score within 10% of the entire network's fitness. The numbers given are for all tasks in aggregate; statistics for individual tasks are not very different. Although we did not select explicitly for simplicity, smaller networks emerged in the simulations. Figure 11 below shows the core network motifs that were evolved for associative learning, identified after pruning.

**Figure 12:**
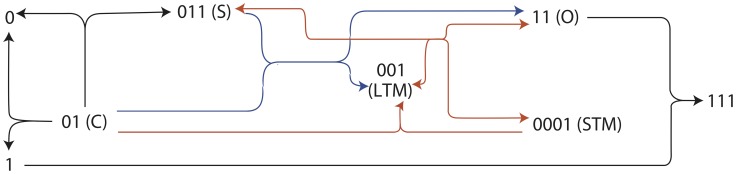
Reactions in the best performing chemical network in the AB-BA task. All reactions are reversible, arrowheads only indicate the thermodynamically favoured direction. S- stimulus, C- control, O- output, STM- short-time memory-trace, LTM- long-term memory-trace. All species decay and there is a low-rate inflow of molecule ‘001’. Blue and red lines correspond to the motifs on Figure 11. In the environment where stimulus pulses are followed by control pulses, output and a short-term memory chemical are produced in response to the stimulus from the long-term memory chemical; then, when the control pulse arrives, the memory chemical is regenerated from the short-term memory chemical.

The second motif (Figure 11A, bottom) is the most commonly evolved solution. It appeared as a solution to all the above tasks. We analyse that in detail below in a case where it evolved in the best network capable of solving the AB-BA task (the network is described in detail in Supporting Information TextS1 part 3). The task in this case is to produce output (species 11) when control pulses follow stimulus pulses (S→C), but not to produce output chemical O when control pulses precede stimulus pulses (C→S), see Figure 12.

In the S→C environment, a slowly decaying long-term memory chemical LTM (chemical species ‘001’) reacts with the stimulus S to produce output O and a fairly rapidly decaying short term memory chemical STM (0001). Thus, output is produced in response to the stimulus when the memory chemical is present:
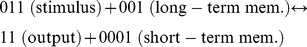
(1)When the control pulse C occurs, it converts the short-term memory chemical back into the long-term memory molecule, allowing the LTM molecule to be reused again in the next pulse pair.
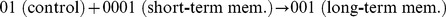
(2)Now consider how the same network must behave quite differently in the C→S condition. Here C occurs before S so there is no STM molecule for C to react with to produce LTM. This means there is no LTM molecule for S to react with to produce output. Instead C readily disintegrates:
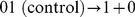
(3)and the disintegration product reacts with the output molecule thus removing any output that might be produced in response to the stimulus that follows:
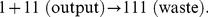
(4)Whilst reactions (3) and (4) are not shown in Figure 11, we note that such ‘extra’ reactions are typical additions to the core motifs that evolved. Each evolved network contains multiple such extra adaptive reactions that help in various ways to control the dynamics of the system.

**Figure 13:**
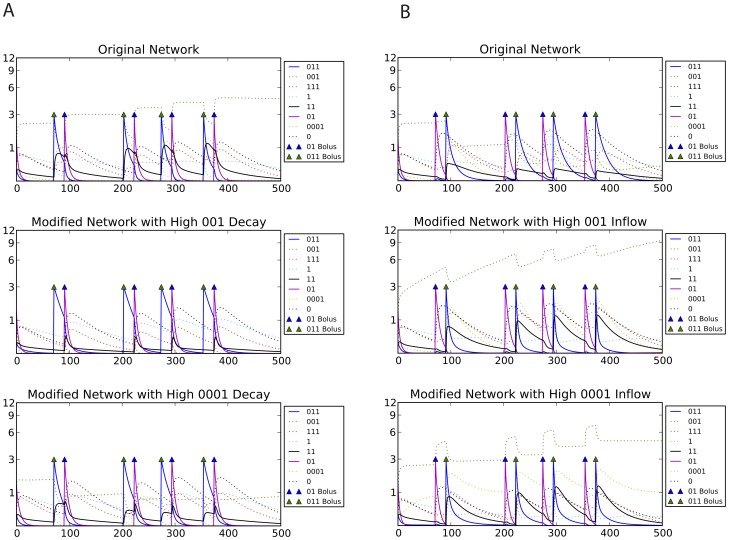
Manipulating the chemical network. Black solid line shows output concentration; blue solid line shows stimulus and purple solid line shows control concentrations. Dotted lines show intermediate chemical concentrations. Triangles show input boluses. In the S→C environment (A) high decay of any of the memory chemicals diminish the response; in the C→S environment (B), high inflow of any of the memory chemicals is enough to produce output.

This hypothesis for the mechanism of learning was tested by modifying the concentration of the long-term and short-term memory chemicals by manipulating their inflow and decay rates and observing the response to stimulus pulses. We found that, as expected, the LTM and STM molecules determined the magnitude of output produced (Figure 13). Remarkably, this explanation can be re-interpreted in the light of Bayesian posteriors, i.e. ‘beliefs’ that the network has about which environment it is likely to be in, according to the information provided so far by the environment. To do this, we interpreted the internal state of the network as encoding a Bayesian posterior, by fitting a regression model from the chemical concentrations of the network at each point in time to the ideal Bayesian posterior of being in the associated environment given the sensory history encountered so far. If it is possible to fit such a regression model it means that a linear combination of chemical species concentrations encodes in a sense a near-optimal ‘belief’ about which environment the network is in. We found it was indeed possible to fit such a linear model for the above network, see Table 2. Furthermore, the parameters of this model must correspond to each species' role in learning. Positive numbers signify chemical species that are typical for the S→C environment, while negative numbers indicate that these chemicals are more abundant in the C→S environment. As expected, the largest positive posteriors belong to the memory chemicals, and, of course, to the output chemical (reactions 1–2); while large negative numbers indicate the disintegration product and the waste chemical (reactions 3–4).

**Table 2:** Coefficients of the regression model that multiply each chemical species concentration to obtain the Bayesian posterior prediction for the best performing chemical network in the AB-BA task.

S→C environment		C→S environment	
Chemical	Weight	Chemical	Weight
011	0.03	111	−1.32
001	1.8	1	−2.38
11	2.57	01	−0.14
0001	1.5		
0	0.81		

Positive numbers indicate species that are more likely to have high concentration in the S→C environments, while negative numbers belong to species that are more prevalent in the C→S environment. The magnitude of the weight relate to the significance of the chemical. The Bayesian interpretation is consistent with our explanation for the learning mechanism (see text).

Many of the evolved networks used the motif described above. There were a few more general features that repeatedly appeared for all tasks. For example, the input (stimulus, control) and output chemicals' concentration typically decreased quickly, either by spontaneous decay or by reactions that converted them to waste products/memory chemicals. A long-term memory chemical could be identified in most networks: this reacted with the stimulus to produce output, and was generated only in the S→C environment.

Apart from these features, the chemical background of learning was diverse and highly specific to the task in question. In the clocked and noisy clocked tasks only the S→C environment contained control pulses, and this was habitually exploited by converting the control directly to the long-term memory chemical (network not shown in Figure 11). In the non-clocked task, many of the networks used the fact that the output needs to be low after the control arrives. The signal in itself was converted to output, while control removed output. This resulted in a dynamics where in the S→C environment, control removed the output of the previous signal; in the case of randomly distributed control pulses, there was no output available when control was added, so, it inhibited the output of the following signal. The AB-BA task was a very special problem and the networks evolved to solve it were even more diverse than usual. In several cases the control was used to inhibit output production, as in the C→S environment it reliably preceded the signal. As the 2-bit task included more environments, it was more difficult for the networks to use “tricks”, and they mostly used the mechanisms depicted on Figure 11. We have evolved a few networks to be able to solve all tasks and the tendency towards simplicity was even clearer in them: they invariably used the most typical mechanism (Figure 11A, bottom) that we have analysed above.

## Discussion

### Bayesian analysis

Bayesian statistics provides a valuable framework, not just for statistical analysis of data, but for conceptualising how physical systems can encode models of their environment and update those models. The central concept in Bayesian statistics is that a “belief” can be modelled as a probability distribution; the rational way to modify the belief in response to evidence can then be formally codified. In order to incorporate cumulative evidence rationally into a model of the environment, it is sufficient to apply Bayes' rule repeatedly over time, with the posterior probability after each observation becoming the prior probability for the next observation, see [24] for an overview. This process is known as iterated (or recursive) Bayesian inference.

The typical application of Bayesian statistics would (in effect) be for the experimenter to apply Bayesian inference to their own beliefs, beginning with some probabilistic belief about the system and refining it by the observation of evidence. We turn this on its head by considering, if the system *itself* were a rational observer, what “beliefs” it should have regarding its environment and how it should update them in response to evidence. A similar approach to ours can be seen in [25]. We found that a Bayesian analysis provides insight into understanding network function. Note that there was no explicit pressure on the networks to perform Bayesian reasoning. However, achieving a high fitness during evolution required the networks to incorporate and integrate information over time. Iterated Bayesian inference is the formal ideal of the process of integrating cumulative evidence; hence, we have a theoretical motivation for interpreting the network dynamics in Bayesian terms.

We attributed “beliefs” to the networks by analytically deriving the Bayesian beliefs (posteriors) of an ideal observer in a given task (over a variety of time steps and environments), and fitting a regression model from the network's state to this ideal belief. (We use a logistic regression model as the natural analogue of a linear model for a range bounded between 0 and 1.) Hence, we determined the maximum extent to which the network's state can be said to encode the correct posterior in a simple form. For comparison purposes, we also performed this procedure on networks that were not evolved on the task in question. This means that the “belief” attributed to a network depended on the task it was being observed on: “belief” in this context really means “most generous attribution of belief given the task”.

**Figure 14:**
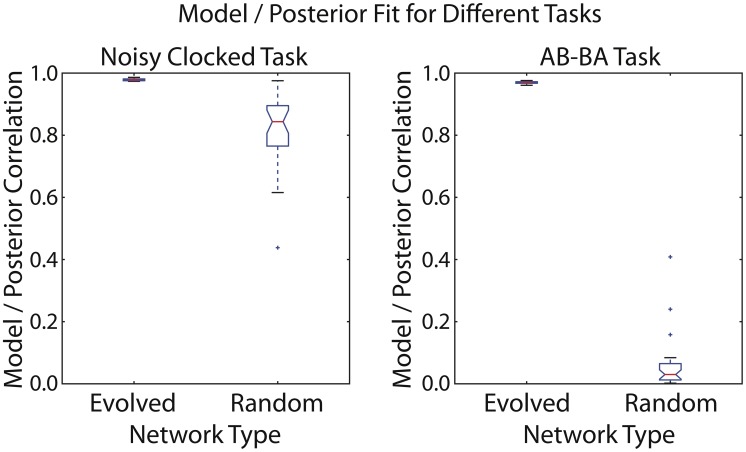
Boxplot showing goodness of fit of a logistic regression model to the ideal Bayesian posteriors in 30 test environments for the noisy clocked and AB-BA tasks. The degree to which a network's state encodes the Bayesian posterior via a logistic model is shown for a single evolved network and 30 random networks.

**Figure 15:**
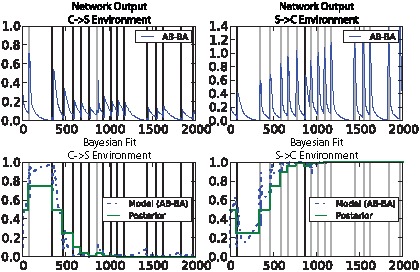
Best “belief” change over time in an evolved network for two paired lifetime runs of the AB-BA task. Upper: network output. Lower: ideal Bayesian posterior (dotted line) and attributed network “belief” based on regression model from concentration values (solid line). Vertical bars illustrate input event timing: dark grey for C→S events and light grey for S→C.

The mean correlation between the fitted logistic regression model and the analytic posteriors is extremely high (0.97–0.98) for the highest-fitness evolved networks on both the noisy clocked association task and the AB-BA task (Figure 14). The information required to perform the noisy clocked task is relatively easy to accumulate in a detectable form: for random networks, the mean model/posterior correlation is fairly high (0.82). For the AB-BA task, which requires accumulating more subtle information, the quality of fit of the regression model for random networks is very low (0.06) (Figure 14). Figure 15 shows the dynamics of an evolved network's best “belief” (the output of the regression model) over time for a particular lifetime, compared to the ideal rational belief (the posterior probability). Interestingly, the network evolved on the “2-bit environment” task demonstrated information capture on both the noisy clocked task (rho = 0.83) and the AB-BA task (rho = 0.73). See Supporting Information TextS1, part 4 for other example networks, including networks that were evolved on a different task to the one they are being tested on.

The process of Bayesian inference is characterised by the incorporation of relevant information into a system's internal state. This does not constrain the way in which a Bayesian posterior is encoded into the state of a system; the encoding in principle could be arbitrarily complex. However, our empirical results for the evolved networks indicate that the existence of an encoding can be demonstrated by a simple regression model.

It is worth observing that just because a system's state contains the relevant information to perform a task, this does not necessarily mean that the system uses that information appropriately. For our noisy clocked task, the dynamics of a randomly constituted network usually encode the relevant information for task performance in a nearly linear way, whereas random networks have a poor fitness performance on the task. This is because in the artificial environment for that task, the overall rate of control pulses differs in the two different experimental conditions. To a first approximation, we can regard the two experimental conditions as providing constant driving inputs to the system, but at different rates. Hence, if a system's gross dynamics depend on the rates of control pulse inputs (which will be true for the majority of systems), then observing the system's state after interacting with one or other of our task environments will readily reveal which environment the system was exposed to. We will see below that this issue does not apply to the more complex AB-BA task that requires genuine sensitivity to stimulus pairing (see Table 1 for a comparison of the informational requirements in the noisy clocked task and the AB-BA tasks).

There are important parallels here to *liquid state machines* [26], [27] and other *reservoir machines* [28] and to *random projections* [29] in machine learning: information capture is not necessarily the hardest part of information processing, and randomly constituted systems can often accumulate information in a usable fashion. So, the random networks store information about the rate of control pulses in the environment (although not as much information as a network evolved for the task). That information can be extracted by an observer using a simple regression model, similar to reservoir machine and random projection learning. However, the random networks do not incorporate the machinery to translate the stored information into an appropriate response: a high output following a stimulus pulse when control pulses have occurred at a high rate in the past, and a low output otherwise.

By contrast, we determine empirically that the AB-BA task produces very different information dynamics to the noisy clocked task. In the AB-BA task, the overall rate of control (and stimulus) pulses is identical in the two different task environments. While random networks can be assigned a logistic-model Bayesian interpretation for the first task (i.e. a regression model can be fitted to map from the network state to the current optimal Bayesian posterior), the same is not true for the AB-BA task (see Supporting Information TextS1, part 4), where only the evolved networks have a good logistic-model Bayesian interpretation. Note that to distinguish the AB-BA environments, the network must respond differently to a C pulse followed shortly by a S pulse than a S pulse followed by a C pulse. The information necessary to distinguish the environments optimally is the relative number of CS versus SC pulses.

A nervous system is not necessary for learning. We have shown that associative learning mechanisms implemented by well-mixed chemical reactions can be discovered by simulated evolution. What differences in principle, then, are there between neurons and chemicals? The key difference between learning in neuronal network and learning in our chemical networks is that in neuronal systems generic learning mechanisms exist that are present at each synapse, irrespective of the particular identity of the pre- and post-synaptic neurons. For example, spike-time-dependent plasticity (STDP) can be found between many neurons. This is possible because neurons share the same genome, and this permits each neuron to express the molecular machinery required for plasticity. On top of this, specificity can be achieved through line labelling, i.e. it is the physical pathway from stimulus to neuron A to neuron B etc. that has meaning, and conveys reference. The capacity to associate arbitrary events X and Y arises when a plastic synapse exists between neurons that represent X and neurons that represent Y.

In our chemical networks, however, there is no modular distinction between chemical species that represent events and the chemical reactions that implement learning. The chemical network for associating X and Y by forming memory-trace M cannot work separately to associate P and Q because of two reasons: (i) the reactor is well mixed and the memory-trace M for X and Y will interfere with the memory-trace M for P and Q (ii) the molecule M will react with X and Y but it cannot without modification react with arbitrary P and Q. In the neural system neither of these constraints exists.

This has important consequences on the scaling properties of neural or chemical systems for associative learning. Suppose that the system needs to be able to learn three independent possible associations (say, A→C, B→C and A→D). The weight (strength) of each association needs to be represented independently in the network, and an associative mechanism implemented to update each weight.

In the neural system this is easy; the associative mechanism is a set of molecules that are expressed in each synapse that implements Hebb's rule or some variant of that rule, which states that events that co-occur have a higher probability of co-occurring in the future. In neuronal systems the weights of the associations are the synaptic strengths. Each neural connection contains the molecular capacity to implement Hebb's rule specifically between distinct neurons. In the chemical system, however, each associative mechanism will be a different chemical pathway, and the pathways will need to be functionally similar while involving species whose chemical properties are distinct (since if the species are too similar, there will be crosstalk between the pathways). In essence, it seems plausible that the chemical system will have to re-implement associative learning independently for every possible association.

We have described chemical networks in this paper that can learn to associate one stimulus with another stimulus. An important qualifier here is that they do not display generic associative learning: the two stimuli that can be associated are genetically specified. Of course, more sophisticated cellular systems such as genetic regulatory networks may be able to overcome the problems we have described. Also, the learning is not independent of timing, but instead the ability of an evolved network to undertake associative learning is greatest for environments where the period between successive stimulus-control pairs resembles that period encountered during evolution, see Supporting Information TextS1, part 5).

We used *in silico* evolution to find small chemical networks capable of carrying out various associative learning tasks. It is often the case that evolution finds solutions that are much more concise, elegant, and parsimonious than would be produced by deliberative cognition. In fact, the simplest chemical network still capable of associative learning consisted of only two chemical reactions. This confirms that there is no reason in principle that associative learning within a lifetime should be confined to multicellular organisms.

So why is the experimental evidence of associative learning in single cells to date equivocal? We are only aware of one experiment that addressed this question [30]. Todd Hennessey showed that aversive classical conditioning occurs in Paramecia. He trained a single paramecium to avoid an electric shock by learning that vibration precedes it. The mechanisms underlying such learning are not known, although it seems possible that voltage gated Calcium channels [31], [32] are involved, perhaps with adenylate cyclase acting as a coincidence detector with cAMP dependent state changes mediating memory as in Aplysia [33], [34]. Similar studies have indicated that other single-celled organisms may have the capacity to learn to associate light and electric shocks [35], [36] although a recent study on individual human immune cells showed habituation but no conditioning [37]. Notice that the task of learning a contingency within a lifetime is entirely different from evolving to respond under an evolutionary regularity that B will regularly follow A in all environments. Whilst there was a recent report that such behaviour is observed in bacteria which anticipate the decrease in oxygen following increase in temperature, these bacteria did not learn to anticipate but rather they evolved to anticipate [38]. Often this critical distinction is not made, resulting in confusion between evolution and learning [39]. To see the difference, note that no bacterium in the above experiment could learn within a lifetime that in some environments increased temperature predicts increased oxygen, whereas in other environments decreased temperature predicts increased oxygen. This association was not learned by a single bacterium, instead, it is an association that was discovered by evolutionary search by populations of bacteria. The very ease with which populations of bacteria and yeast can evolve to anticipate environmental changes in laboratory evolution experiments suggests that it may simply not have been necessary for individual single celled organisms to learn to anticipate within a lifetime [40].

An important implication of our work is that the associative mechanisms we have described may be active during development in cells within a multicellular organism. It will be of interest to use bioinformatics to examine whether the motifs in Figure 11 can be found in regulatory networks involved in development. This paper allows us to re-examine the possible function of simple chemical motifs within an associative learning framework.

## Methods

### Artificial chemistry

In order to enforce conservation of atomic mass in the networks' reactions, we used a combinatorial abstract chemistry for the networks. Each simulated chemical species had a “formula” consisting of a string of digits representing chemical “building blocks”, and reactions were constrained to conserve building blocks. These constraints were modelled using three different abstract combinatorial chemistries: An “aggregate” chemistry, where only the number of digits (and not their sequence) determined the species' identity, somewhat resembling inorganic chemistry with atoms as building blocks. Any interchange of building blocks was allowed to happen in reactions. A “rearrangement” chemistry, where the sequence of digits characterized species, somewhat resembling organic chemistry with atomic groups as building blocks. Any interchange of building blocks was allowed to happen in reactions. A “polymer” chemistry, where only ligation and cleavage reactions could happen among chemical species, resembling polymer reactions with monomers as building blocks.

Simulations of a simple aggregation chemistry provided chemical networks with the highest fitness (Supporting Information TextS1, part 6), thus, results in the main text refer to this particular chemistry. Reactions were modelled reversibly. We incorporated further thermodynamic constraints by assigning “free energy” values to chemical species; these constrained the ratios between forward and reverse reaction rates. Each network received an inflow of one particular chemical type (“food”), and every chemical species exhibited first-order decay, as expected in a flow reactor scenario. Note that although all the parameters of the reaction networks – chemical species, chemical reactions, free energy values, inflow rate of food and species decay rates – were allowed to change during the evolutionary runs, each individual network had its own fixed chemistry that stayed the same during the learning trials. Therefore the difference between chemical networks in the unassociated and associated environments could only be induced by the different history of input boluses; these must have modified the state of the network (the concentration of different chemicals) so that it showed different behaviour when presented with the stimulus chemical.

### Encoding

**Figure 16:**
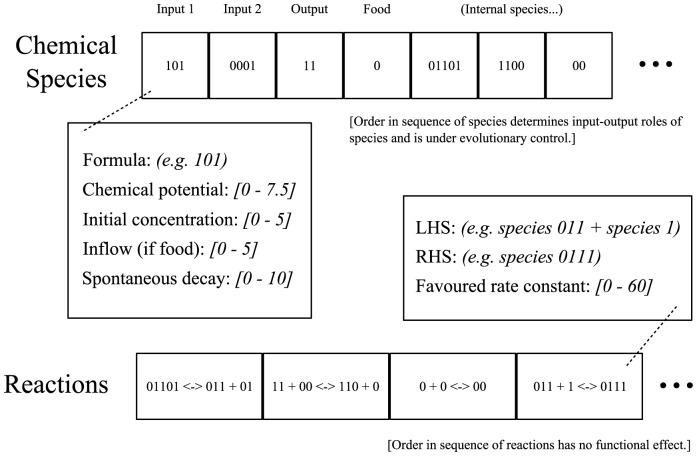
Network genotype and its meaning. For explanation see text.

Networks consisted of a number of chemicals and reactions, the relevant characteristics of which were encoded genetically. See Figure 16 for illustration.

### Chemicals

Each abstract chemical species was associated with a number of real-valued parameters: A chemical “potential”, which affected the thermodynamics of the system, an initial concentration, a spontaneous decay rate (conceptualised as decay to inert waste products), an inflow rate if this species was chosen as the network “food” (see below). In addition, chemical species were assigned a binary “formula” string, which constrained how different species could combine (see “chemistry” section).

### Reactions

Reactions were represented as a list of one or two “Left Hand Side” (LHS) species, a list of one or two “Right Hand Side” (RHS) species, and a real- valued “favoured rate constant” (see below). The variation operators used in evolution guaranteed that reactions conserved mass and compositional elements (see below). Note that the intrinsically favoured direction for the reaction was not determined by the reaction's encoding but by the chemical potential values of the species involved. The “favoured rate constant” parameter of the reaction determined the rate constant in the favoured direction; the rate constant in the non-favoured direction was determined by the chemical potential values of the species involved.

### Input and output

The choice of which chemical species the network used as input, output and “food” were under evolutionary control. Part of the network encoding was an ordered list of species: the first species in the list functioned as inputs; the next species as output; the next species as “food”; and the remainder had no special environmental significance, see Table 3.

**Table 3:** Allowable range, initialisation range, and description for real-valued network parameters.

Chemical Species Parameter	Range	Description
Chemical potential	0–7.5 units	Parameter affecting reaction rate constants
Initial concentration	0–5 units (initialised 0–2)	Concentration of species at the start of a protocol simulation
Inflow (if food)	0–5 units (initialised 0–1)	Inflow rate of the species if selected as the “food” species
Spontaneous decay	0–10 units (initialised 0–1)	Decay rate of the species

Reaction Parameter	Range	Description
Favoured rate constant	0–60 units (initialised 0–0.1)	Rate constant of the reaction in the thermodynamically favoured direction (determined by potentials of reactants).

### Variation

Network mutations were implemented as follows, based on a mutation rate sigma: All real-valued parameters were mutated by Gaussian noise, with reflection at the upper and lower parameter limits. The standard deviation of the noise was scaled by the product of sigma with the absolute size of the allowable range for that parameter. With probability sigma * 5, the program attempted to add a random new reaction to the network (see “adding new reactions”). With probability sigma * 5, a uniformly chosen reaction was deleted from the network. With probability sigma, two elements of the input-output list for the network were randomly swapped (most of the time, this involved swapping “non-special” elements and had no functional effect).

### Adding new reactions

When a mutation called for adding a new reaction to the network, one of the following three possibilities was chosen uniformly:

1. A reaction decomposing an existing chemical species into two molecules. If this was impossible (i.e. the chosen species had a “1” or “0” formula), no reaction was added
2. A reaction composing two existing chemical species into a single molecule. If this would produce “too long” a molecule, a reaction of the third type was generated instead.
3. A reaction rearranging two existing chemical species into two different species. This was modelled as composition followed by decomposition.

In each case, the existing species were chosen uniformly and formulas for the reaction products were generated according to the current chemistry (see “chemistries”). If a formula was generated in this way that did not match a species already in the network, a new species was generated with that formula and added to the network. When a new reaction was added to the network, its “favoured rate constant” parameter was initialised to a low value (uniformly in the range [0, 0.1]) to allow for relatively neutral structural mutations.

### Chemistries

Each chemical species in a reaction network was given a binary string “formula” which constrained what products it could form with other species. Reactions were always constrained so that the total number of 0s on the reaction LHS was the same as the total number of 0s on the RHS, and similarly for 1s. In addition, we modelled three different string “chemistries”, each with different compositional rules, see Table 4.

1. A “polymer” chemistry, where composite formulas involved only concatenation, e.g. 01+00↔0100.
2. A “rearrangement” chemistry, where composite formulas could have their binary elements in any order, e.g. 01+00↔0001 or 0010 or 0100 or 1000. Composition here was implemented as concatenation followed by fair shuffling of string characters.
3. An “agglomeration” chemistry, where only the total number of 0 s and 1 s in a formula (and not the order of them) distinguished different species, e.g. 01+011↔00111. Composition here was implemented as concatenation followed by lexicographic sorting of string characters.

**Table 4:** Composition and decomposition operators for three different types of network chemistry.

Chemistry	Composition	Decomposition
Polymer	“Gluing” one string to the end of the other (*concatenation*), e.g. 011+01→01101	String division at a uniformly chosen location guaranteed to respect maximum string length of products (*splitting*), e.g. 0110101→0110+101
Rearrangement	Concatenation, followed by order randomisation of characters (*shuffling*), e.g. 011+01 (via 01101)→10011	Splitting of shuffled string, e.g. 0110101 (via 1011001)→101+1001
Aggregation	Concatenation, followed by lexicographic reordering of characters in product string (*sorting*), e.g. 011+01 (via 01101)→00111	Splitting of shuffled string, followed by sorting of each product string. e.g. 0001111 (via 1011001) (via 101+1001)→011+0011

### Initialising networks

Networks were initialised as follows. A small number of “seed” chemicals (by default, 4) with distinct formulas of length 3 were added to the network. New chemical species, whether generated at initialisation or due to adding a new reaction to the network during initialisation or mutation, were initialised with uniformly random parameters in the following ranges: potential [0–7.5], initial concentration [0–2], food inflow [0–1], decay [0–1]. The function to add a new reaction was called 20 times, thereby adding an unpredictable number of new chemicals to the network. New reactions, whether generated during initialisation or mutation, were initialised with a uniformly random “favoured reaction constant” in the range [0–0.1]. The input-output list for the network was shuffled fairly.

### Evolution

The networks were evolved using a non-generational genetic algorithm (GA) similar to the Microbial GA [41]. A genetic algorithm is the natural selection algorithm run in a computer [10], specifically it is artificial selection in which an explicit fitness function (phenotypic target) is defined, rather than allowing fitness to emerge as the result of ecological interactions. In our case the fitness function rewards chemical networks capable of the kind of associative learning we require. The basic algorithm was as follows:

Initialise a population with a given number of networks
For a fixed number of iterations,
Pick two different networks from the population (for spatial evolution, choose two neighbours)
Evaluate both networks
Replace the worse-performing network with a mutated copy of the better-performing network

### Simulation

All reactions were modelled using reversible deterministic mass action kinetics (apart from the implicit decay reactions which are irreversible). It is clearest to explain this scheme by example.

A single reversible reaction can be conceptually split into two parts, so that

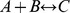
 (with forward rate constant 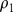 and reverse rate constant 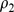)

is conceptually equivalent to the composition of two reactions

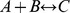
 (with rate constant 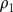)

and

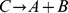
 (with rate constant 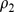)

The rate at which a reaction takes place, in our simulation, is set equal to the product of the concentrations of those species on its left-hand side, multiplied by its rate constant. The reaction consumes its reactants at this rate and generates its products at this rate. The overall rate of change of a species' concentration due to explicitly-modelled reactions is equal to the sum of the rates at which it is generated (over all reactions) minus the sum of the rates at which it is consumed (over all reactions). Spontaneous decay (at a rate 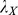 for chemical X) contributes an additional 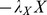 term to this sum, and inflow (at rate 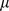 for the food chemical F) contributes an additional 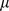 term to F's rate of change. Hence, a system consisting only of the reaction
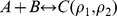
(with A as the food chemical) has the following differential equations:
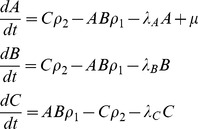
For computational efficiency, simulations during evolution used Euler integration with a step size of 0.01 time units. Input boluses were modelled as discontinuous jumps in concentration at the given time steps. These simulations were qualitatively validated after evolution using a variable step-size Runge-Kutta ODE solver.

### Task protocols

Networks were simulated on chemical protocols, with each protocol consisting of a time series of input boluses, and a time series of target values for the network output. Note that for most time steps, the input bolus values were zero and the target output values were “don't care”. The exact details of the protocol inputs and targets varied from task to task.

### Fitness evaluation

For every task, networks were simulated on a number of protocols, and the (instantaneous) concentration of the designated network output chemical compared to the protocol target for every time step. The fitness of a network was set equal to the negative mean square difference between these two quantities averaged over all protocols and all time steps (ignoring time steps where a “don't care” target was specified). In order to provide a reliable fitness comparison, when two networks were chosen for competition during evolution, they were evaluated on the same set of protocols. Additionally, the protocols for different experimental conditions within the same task were deliberately matched to be similar, so that network response to the experimental condition could be measured as directly as possible.

### Task descriptions

Initial experiments indicated that randomly generating protocols during evolution results in very noisy fitness comparisons, with little fitness gradient for evolution to climb. To avoid this problem, for each task we generated fixed “training data” and saved it to file. Networks were evaluated during evolution on their performance on the training data set. For most tasks, the training data set was a file consisting of 10 randomly generated protocols. A number of tasks were devised requiring the detection of different environmental features by the networks. Some of these tasks were “clocked”, i.e. pulses were constrained to only occur at predetermined regular “clock tick” times, and some were not.

### Clocked task

This task constrained B boluses to a regular “clock tick” schedule every 100 time steps and had two experimental conditions. There was only a 0.5 probability of a chemical B bolus on a given clock tick. In the “associated” condition, a chemical B bolus was always followed 20 time steps later by a chemical A bolus. In the “unassociated” condition, chemical A boluses never occurred. A single protocol featured both experimental conditions, with identical B boluses in each condition. The desired behaviour for the network was: upon receiving a pulse of chemical B, output either zero (in the “unassociated” condition) or one (in the “associated” condition) for 20 time steps afterwards.

### Clocked task with noise

This was identical to the previously described task except that there was a small (p = 0.1) probability of “noise” occurring at each time step with a chemical B bolus. Noise consisted of a B bolus being followed by an A bolus in the “unassociated” condition or a B bolus followed by no A bolus in the “associated” condition. Within a single protocol, the occurrence of noise was matched between experimental conditions.

### Non-clocked task

This task had two experimental conditions and involved boluses at random intervals. In both conditions, pulses of chemical B occurred at random intervals uniformly in the range [100, 300]. In the first (“associated”) condition, a pulse of chemical B was followed shortly afterwards (20 time steps) by a pulse of chemical A. In the second (“unassociated”) condition, pulses of chemical A occurred independently of B, at random intervals uniformly in the range [100, 300]. Within a single protocol, pulses of chemical B were identical.

### AB-BA task

This task, featuring two experimental conditions, was specifically designed to involve a non-trivial accumulation of information. Within this task, input “events” occurred randomly at a low rate (0.025 per time step) with a refractory period of 50 time steps between events, over a total period of 2000 time steps. Each event consisted of either a pulse of chemical A followed closely (20 time steps later) by a pulse of chemical B, or vice versa. In the first experimental condition (“A→B”), events were 75% likely to be “A→B” pulses and 25% likely to be “B→A” pulses, and vice versa for the second (“B→A”) experimental condition. The desired output behaviour was to respond to a “B” pulse with a low output in “A→B” environments and a high output in “B→A” environments. Note that this task was both noisier than the other tasks and involved a longer evaluation period (to allow the noise some time to average out).

### 2-Bit environment task

Unlike the other tasks, every environment in this task was designed by hand. The intention was to construct a range of radically different environments such that both short- and medium- term network memory-traces would be required to attain maximum fitness. The inspiration was loosely drawn from the concept of the “radical envelope of noise” [Jakobi, 1998]. Input pulses (boluses) in this task always occurred in closely-separated pairs, although the second bolus in a pair did not have to contain the same chemical as the first bolus. The pulse pairs occurred at regular intervals of 100 time units each. Each experimental condition was characterised by a “typical” pulse pair (A→A, A→B, B→A or B→B). In addition to the “typical” pulse pair corresponding to the experimental condition, every protocol for this task also had a “noise” pulse pair. There were in total 4 protocols (one for each pulse pair type), each containing 4 experimental conditions, for a total of 16 different input series. A single input series had the following structure: First, a pulse pair corresponding to the protocol's “noise” pair. Next, three “signal” pulse pairs all of the “typical” type for that experimental condition. Next, a “probe” pulse pair (see below). Next, another “noise” pulse pair of the protocol's “noise” type. Last, a final “probe” pulse pair. “Probe” pulse pairs consisted of a pulse of “B” chemical followed by either a pulse of “A” chemical (in the B→A environment) or a pulse of “B” chemical (in other environments). The desired network behaviour was to produce a low output for 10 time steps prior to each “probe” pulse pair, followed by either a high output (in the B→A environment) or a low output (in other environments) for 20 time steps. Errors in the B→A environment were weighted three times as heavily as errors in the three other environments.

### Network connection density

We calculate the number of reactions per effective chemical species in a network by first excluding any species which do not take part in reactions (this is possible if all reactions featuring a particular species are lost from a network by structural mutation). We then simply calculate the mean number of distinct reactions each remaining species is involved in.

To investigate the effects of different genetic encoding factors on network connection density, we conducted 10 evolutionary runs on the 2-bit environment problem in each of 4 encoding variations. These were:

1. A benchmark case with maximum formula length 4, 2 symbols in the chemical alphabet, and the aggregation chemistry.
2. A variation of the benchmark case with maximum formula length 6.
3. A variation of the benchmark case with 4 symbols in the chemical alphabet.
4. A variation of the benchmark case using the rearrangement chemistry.

For all these runs, we recorded the effect of every mutation on both fitness and also the number of reactions per chemical species.

### Bayesian interpretation of evolved networks

Our method is as follows. We imagine an ideal Bayesian reasoner, equipped with knowledge of the statistics of the different network task environments. For each input train, at each point in time, we calculate what subjective probability the reasoner should assign to the possibility that the input train up to that point came from an “associated” environment. This establishes what the ideal Bayesian posterior would be at each point in time for each input train. If a network's chemical concentrations somehow encode this time-varying Bayesian posterior in all environments, then it would seem reasonable to attribute a Bayesian interpretation to the network. For the purposes of this paper, we will skirt over the complexities introduced by the non-dissipation of information in smooth continuous dynamical systems. In principle, the state of our simulated networks will usually contain all information about their historical inputs, because information can be stored in arbitrarily small differences in concentrations. However, in practice this information will be destroyed by noise.

### Calculating ideal posteriors

Calculation of the ideal posteriors for our environments is straightforward. A random variable X will represent the type of environment: either 1 (“associated”) or 0 (“unassociated”). Another random variable *Y(t)* will represent the train of input boluses up to time *t*. The ideal posterior probability of being in the “associated” environment after observing an input train *y* is
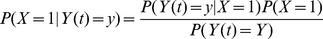
where
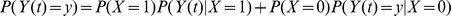
The prior *P(X = 1)* was set equal to the proportion of “associated” environments in the network training sets, i.e. 0.5. The probabilities *P(Y(t) = y|X)* were calculated as follows. In the environments we analysed in this way, Input trains were organised into “events”: a bolus of one or other input chemical, followed possibly at a set short interval by another bolus. The time between input events always exceeded the inter-spike interval within an event. The timing of events in an input train provided no information about the type of environment. Hence, events can be extracted from an input train and treated as a discrete process. “Associated” and “unassociated” environments correspond to Bernoulli processes with “associated” (B→A) and “unassociated” (B alone, for the noisy clocked task, or A→B for the AB-BA task) events. Thus, the posterior *P(X|Y(t))* can be calculated by counting the total number of associated and unassociated events in *Y(t)*.
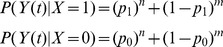
where *n* and *m* are the number of “associated” and “unassociated” events in *Y(t)*, and *p_1_* and *p_0_* are the probabilities of an “associated” event in the “associated” and “unassociated” environments respectively. This gives
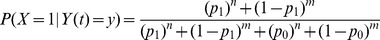
which can be attached to a time series at the appropriate points after events have occurred.

### Matching posteriors to network state

We use a straightforward logistic regression model to match network concentrations to Bayesian posteriors. Given a concentration vector *x*, a weight vector *w* and a bias value *b*, the model is
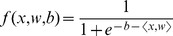
where 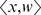 is the scalar product of *x* and *w*. Note that the output of this function is bounded between 0 and 1 like a probability value (this would not be the case for a linear regression model). The idea is that we can investigate the degree to which a rational Bayesian belief is encoded transparently in the network's state. We expect that at time *t*, having observed an input history *Y(t)*,
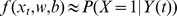
To determine appropriate model parameters, we randomly generate 200 environments (100 “associated” and 100 “unassociated”), and run the evolved network in those environments. Weights w and bias b are set to minimise mean square error over all environments and time steps, using Levenburg-Marquadt optimisation. No attempt was made to regularise the parameters or otherwise avoid overfitting, since the model has relatively few parameters. For comparison, 200 random networks (produced by random initialisation followed by 200 mutations) were tested in the same way.

## Supporting Information

Text S1 Supporting information file.
(DOCX)
