# Supplementary material for: Evolution of Associative Learning in Chemical Networks
Source: PLoS Comput Biol. 2012 Nov 1;8(11):e1002739. doi: 10.1371/journal.pcbi.1002739 (PMC3486861; doi:10.1371/journal.pcbi.1002739)
Supplement: Text S1 — Supporting information file. (DOCX) [file pcbi.1002739.s001.docx]

Supporting Information File 1

***Part 1. Previous Network Experiments***

Prior to the evolutionary experiments described in the main body of this paper (the “V2” experiments), we ran a number of similar experiments under less realistic conditions (the “V1” experiments). Space does not permit the description of these experiments in detail, but we summarise them in this appendix. A number of methodological differences pertained, with the main ones being:

- V1 experiments modelled inputs to the networks as externally-maintained fixed concentrations of input chemical. Inputs on the V1 tasks tended to overlap in time. V2 experiments modelled inputs as boluses which did not overlap.
- Initial concentrations for V1 networks were fixed, whereas for the V2 networks they were under evolutionary control.
- V1 networks had a designated “waste” chemical with a very low potential energy.
- V1 networks used much less realistic constraints on what reactions were permissible. The only constraint on V1 reactions was that each reaction should have exactly two reactants and exactly two products. By contrast, the V2 experiments assigned a compositional “formula” to each chemical species, and used these formulas to determine what reactions were possible.
- V1 networks had a fixed number of reactions and chemical species. V2 networks could grow or shrink during evolution.

The networks evolved in the V1 experiments were significantly simpler than the V2 networks described in the main body of this paper, sometimes comprising as few as four chemicals and two reactions after automated pruning. Analysis of the evolved networks showed that they often used reversible reactions to perform more than one function (e.g. both response formation and memory decay). This was usually only possible because levels of input chemicals were actively regulated by the environment.

## Example of V1 Evolved Networks

For instance, Figure S.1.1 shows the reactions in a simple evolved V1 associative learning network, after pruning the reactions and chemicals which contributed little to network fitness. This network produces an elevated output chemical concentration in response to input B, providing that in the past input B has tended to be paired with input A. The central mechanism involves a “memory” chemical, functioning as an output precursor, which is elevated over time when A and B co-occur, and depleted over time when B occurs alone.

This network works as follows (Figure 1.2): under conditions of high B (stimulus) and low A (control), the memory chemical is converted into output (by reaction with B), and B catalyses the decay of the output (into A, which is actively removed from the system by the environment). Under conditions of high B and high A, a rapid production of output occurs (from A, catalysed by B) and the output is partially converted into memory chemical (and excess B, which is removed by the environment). In the absence of B, output rapidly decays into memory (and B, which is removed). Hence, repeated B inputs (stimulus) in the absence of A (control) produce progressively smaller outputs, while B inputs (stimulus) which overlap to some degree with A inputs (control) produce progressively larger outputs.


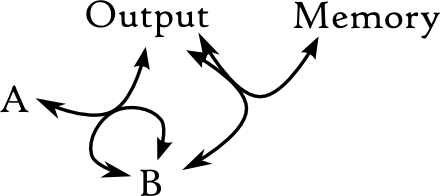


Figure S.1.1. Example of a four-species, two-reaction network for simplified associative learning.


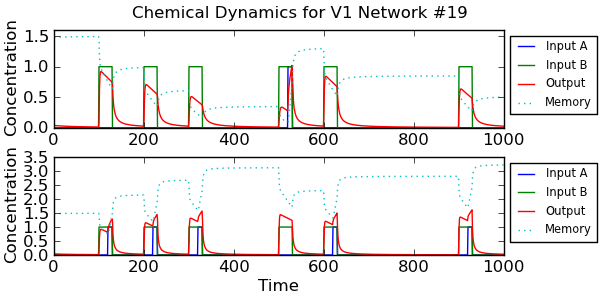


Figure S.1.2. Sample dynamics of the network shown in Figure S.1.1. Concentrations of input A (control), input B (stimulus), output chemical and the intermediate “memory” chemical are shown over time. Above: unassociated condition. Below: associated condition.

## General Findings

We were able to produce a large number of networks which performed the same task as the one described in section 1.1, and analyse their core dynamics.

### Path from stimulus to output

The majority of V1 evolved networks used a mechanism where the primary path from input B to output involved the reversible conversion of a single intermediate memory chemical. When B was being supplied, the memory would react with B to produce output; when B was being removed from the system the output chemical would reconvert to memory chemical using the same reaction.

In about 20% of the networks, the reaction producing output from memory and B was effectively irreversible, and the reconversion from output to memory used a different pathway. A few networks used other mechanisms, such as a memory which catalysed the production of output from B. (See Figure S.1.3 for examples and a breakdown of different path types.)


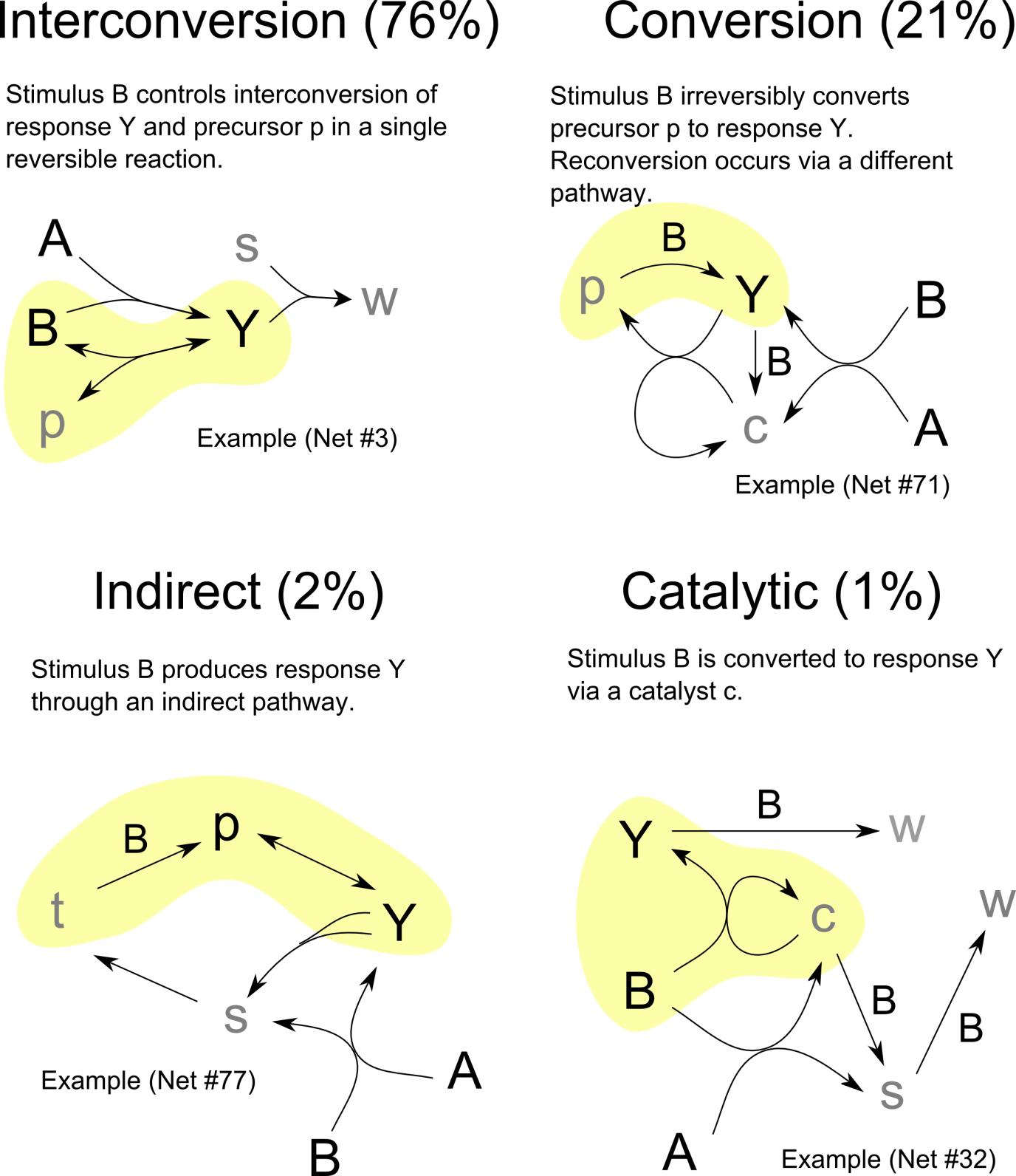


Figure S.1.3. Four path types from stimulus (B) to response (Y) in 100 V1 evolved networks, with examples.

### Memory Formation

#### Associative or Simple Learning

The task the V1 networks were evolved on could be accomplished without performing associative learning. Some networks did exactly this, forming a memory of the occurrence of input A alone, without any mechanism to relate it to input B. However, these networks were in the minority (~20%). Most of the networks (~80%) incorporated a genuine associative learning mechanism, with a reaction between A and B detecting the co-occurrence of inputs (see Figure S.1.4 for illustrative examples).


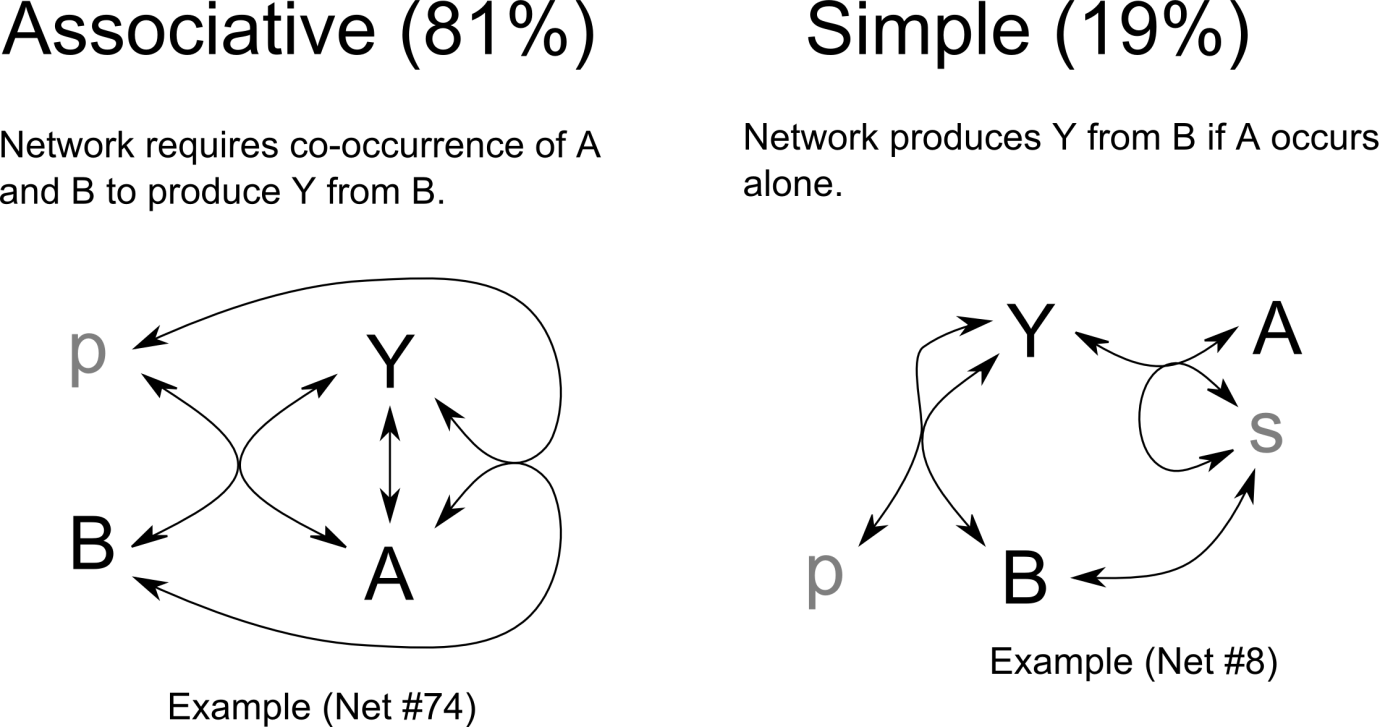


Figure S.1.4. types of learning (associative or simple) in 100 V1 evolved networks, with examples.

#### Memory Formation Pathways

Whether learning was associative or simple, most networks primarily stored their memory of previous inputs in a single chemical species p which facilitated production of the output Y (either as a precursor or a catalyst). There was accordingly a pathway allowing supply of the control (A) input to result in increased concentration of p, since the networks were evolved to produce Y only when their past history included high A inputs. This pathway was usually indirect, involving Y itself, although often it included a direct pathway from A to p. Figure S.1.5 describes the variations we observed, with examples.


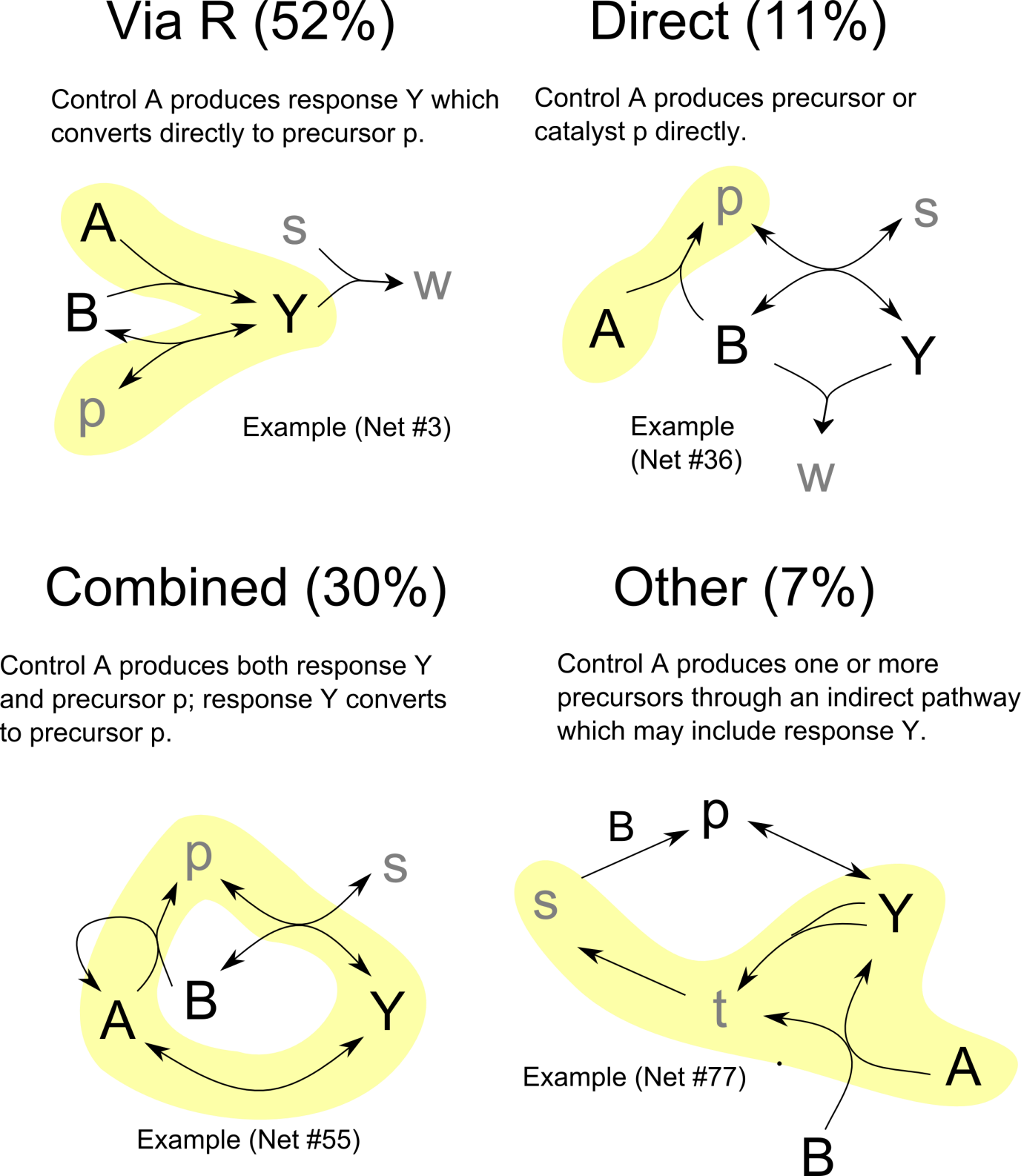


Figure S.1.5. Path types from control B to memory p in 100 evolved V1 networks, with examples.

***Part 2. Extinction***

All the evolved networks presented in the paper are capable of extinction, see Figure S.2.1 to S.2.5 below.


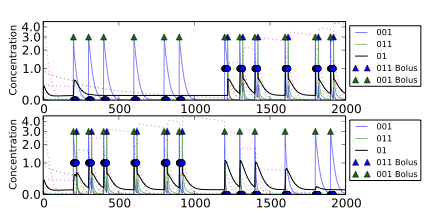


**Figure S.2.1 showing reversible learning dynamics for the clocked task. Above: 1000 time steps of "unassociated" condition followed by 1000 time steps of "associated" condition. Below: 1000 time steps of "associated" condition followed by 1000 time steps of "unassociated" condition. Black solid line shows output concentration; blue solid line shows stimulus concentration; green solid line shows control concentration. Dotted lines show intermediate chemical concentrations. Circles indicate notional target output values for the network. Triangles show input boluses.**


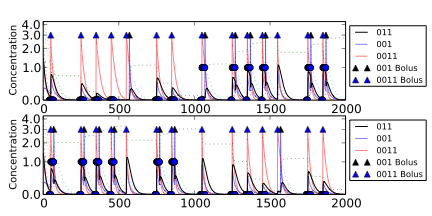


**Figure S.2.2 showing reversible learning dynamics for the noisy clocked task. Above: 1000 time steps of "unassociated" condition followed by 1000 time steps of "associated" condition. Below: 1000 time steps of "associated" condition followed by 1000 time steps of "unassociated" condition. Black solid line shows output concentration; red solid line shows stimulus concentration; blue solid line shows control concentration. Dotted lines show intermediate chemical concentrations. Circles indicate notional target output values for the network. Triangles show input boluses.**


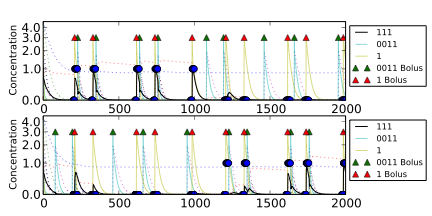


**Figure S.2.3 showing reversible learning dynamics for the non-clocked task. Above: 1000 time steps of "unassociated" condition followed by 1000 time steps of "associated" condition. Below: 1000 time steps of "associated" condition followed by 1000 time steps of "unassociated" condition. Black solid line shows output concentration; yellow solid line shows stimulus concentration; blue solid line shows control concentration. Dotted lines show intermediate chemical concentrations. Circles indicate notional target output values for the network. Triangles show input boluses.**


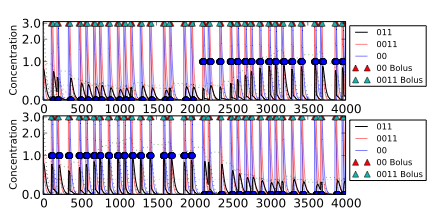


**Figure S.2.4 showing reversible learning dynamics for the AB-BA task. Above: 1000 time steps of "unassociated" condition followed by 1000 time steps of "associated" condition. Below: 1000 time steps of "associated" condition followed by 1000 time steps of "unassociated" condition. Black solid line shows output concentration; red solid line shows stimulus concentration; blue solid line shows control concentration. Dotted lines show intermediate chemical concentrations. Circles indicate notional target output values for the network. Triangles show input boluses.**


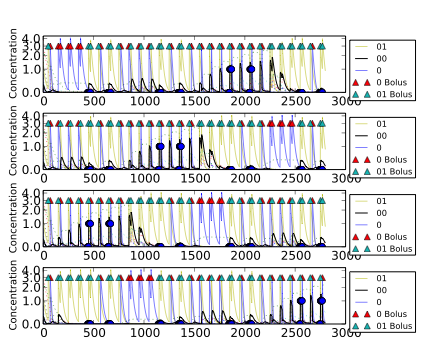


**Figure S.2.5 showing reversible learning dynamics for the 2-bit task. The four time series show a loop of consecutive C->C, C->S, S->C and S->S environments, started at different points in the cycle. Black solid line shows output concentration; yellow solid line shows stimulus concentration; blue solid line shows control concentration. Dotted lines show intermediate chemical concentrations. Circles indicate notional target output values for the network. Triangles show input boluses.**

*Part 3. Best performing networks for each task*

**CHAMPION NETWORK – CLOCKED TASK**

Fitness -0.184576 (non-pruned)

== REACTIONS ==

000 <-> 0 + 00 [ k-> = 36.145961, k<- = 0.718663 ]

000 + 0011 <-> 001 + 0001 [ k-> = 0.063361, k<- = 0.001476 ]

001 + 011 <-> 0011 + 01 [ k-> = 25.894759, k<- = 0.048347 ]

01 + 01 <-> 0011 [ k-> = 16.858558, k<- = 14.660408 ]

00 + 01 <-> 0001 [ k-> = 6.469377, k<- = 0.169316 ]

011 + 0011 <-> 001 + 0111 [ k-> = 31.534313, k<- = 21.501031 ]

01 + 011 <-> 0 + 0111 [ k-> = 21.921524, k<- = 0.333969 ]

000 + 1 <-> 0001 [ k-> = 7.239927, k<- = 0.000074 ]

111 + 011 <-> 11 + 0111 [ k-> = 23.652974, k<- = 0.087727 ]

== SPECIES ==

001 (start 0.004218, decay 2.721381)

011 (start 0.155827, decay 3.738387)

000 (start 1.617242, decay 3.626139)

0 (start 4.278178, decay 4.204123)

01 (start 0.045266, decay 0.873102)

00 (start 0.866823, decay 1.552885)

0001 (start 0.628683, decay 1.969112)

1 (start 1.349662, decay 5.946280)

0011 (start 0.669255, decay 3.171469, inflow 0.372868)

0111 (start 0.283833, decay 0.047865)

11 (start 0.255579, decay 3.106958)

111 (start 1.008871, decay 0.217617)

== INPUTS / OUTPUTS ==

Input(s)

control 011

stimulus 001

Output(s) 01**CHAMPION NETWORK – NOISY CLOCKED TASK**

Fitness -0.223420 (non-pruned)

== REACTIONS ==

1 + 1 <-> 11 [ k-> = 27.134344, k<- = 0.000023 ]

1 + 0011 <-> 01 + 011 [ k-> = 16.688289, k<- = 0.001433 ]

00 + 0011 <-> 01 + 0001 [ k-> = 2.552790, k<- = 0.000384 ]

1 + 11 <-> 111 [ k-> = 35.550818, k<- = 0.024558 ]

1 + 11 <-> 111 [ k-> = 20.060266, k<- = 0.013857 ]

011 + 001 <-> 01 + 0011 [ k-> = 37.786091, k<- = 14.190899 ]

0011 + 111 <-> 011 + 0111 [ k-> = 53.336543, k<- = 20.921865 ]

1 + 1 <-> 11 [ k-> = 24.144040, k<- = 0.000020 ]

001 + 001 <-> 01 + 0001 [ k-> = 8.079743, k<- = 0.065505 ]

0 + 001 <-> 0001 [ k-> = 3.015471, k<- = 0.272376 ]

001 <-> 0 + 01 [ k-> = 3.437779, k<- = 0.308559 ]

== SPECIES ==

011 (start 1.296289, decay 1.833331)

001 (start 0.121179, decay 3.031684)

111 (start 0.702826, decay 6.130618)

1 (start 1.484043, decay 2.271954, inflow 0.004331)

11 (start 2.001847, decay 1.662993)

01 (start 0.478344, decay 0.005378)

0011 (start 2.059817, decay 0.989031)

00 (start 4.236181, decay 3.516769)

0001 (start 4.231879, decay 2.921251)

0 (start 3.057655, decay 7.834180)

0111 (start 4.165371, decay 1.612433)

== INPUTS / OUTPUTS ==

Input(s)

control 001

stimulus 0011

Output(s) 011 **CHAMPION NETWORK – NON-CLOCKED TASK**

Fitness -0.160684 (non-pruned)

== REACTIONS ==

111 + 01 <-> 11 + 011 [ k-> = 50.495125, k<- = 6.218730 ]

1 + 11 <-> 111 [ k-> = 30.474017, k<- = 0.085481 ]

0011 <-> 01 + 01 [ k-> = 56.801240, k<- = 26.278366 ]

0 + 001 <-> 0001 [ k-> = 27.250181, k<- = 0.041249 ]

011 + 01 <-> 00 + 111 [ k-> = 28.673850, k<- = 18.431607 ]

000 + 001 <-> 00 + 0001 [ k-> = 14.909582, k<- = 5.013260 ]

11 + 01 <-> 0111 [ k-> = 0.165339, k<- = 0.003481 ]

1 + 00 <-> 001 [ k-> = 7.968099, k<- = 0.116654 ]

0 + 0011 <-> 00 + 011 [ k-> = 16.005928, k<- = 0.000099 ]

01 + 11 <-> 0111 [ k-> = 0.143812, k<- = 0.003028 ]

1 + 00 <-> 001 [ k-> = 0.018624, k<- = 0.000273 ]

== SPECIES ==

011 (start 0.154024, decay 5.366232)

111 (start 1.105991, decay 4.227602)

001 (start 4.047437, decay 3.394207)

11 (start 0.288689, decay 0.050129)

0011 (start 1.508115, decay 4.414706)

01 (start 1.093057, decay 4.474895)

1 (start 3.697918, decay 7.725799)

0 (start 0.748894, decay 4.815533)

00 (start 4.513877, decay 8.277158)

0111 (start 4.697246, decay 2.031167, inflow 1.694810)

0001 (start 2.697954, decay 3.559942)

000 (start 1.201312, decay 2.561234)

== INPUTS / OUTPUTS ==

Input(s)

control 0011

stimulus 1

Output(s) 111

**CHAMPION NETWORK – AB-BA TASK**

Fitness -0.238285 (intact)

== REACTIONS ==

1 + 11 <-> 111 [ k-> = 12.52907777600140803997, k<- = 2.28166407031307993947 ]

01 + 0001 <-> 001 + 001 [ k-> = 45.23201636935901603920, k<- = 0.00280666589480160983 ]

01 + 01 <-> 0 + 011 [ k-> = 0.47775513290856785886, k<- = 0.00041445787838336238 ]

1 + 11 <-> 111 [ k-> = 19.73746266086768130776, k<- = 3.59437942660934162120 ]

01 + 011 <-> 11 + 001 [ k-> = 11.59922643479641735098, k<- = 0.00040766490583340169 ]

001 + 011 <-> 11 + 0001 [ k-> = 4.99592169962727172816, k<- = 2.82973100895890672390 ]

01 <-> 1 + 0 [ k-> = 10.26907387169977070585, k<- = 0.07371542360272706484 ]

== SPECIES ==

011 (start 0.24817417373871911157, decay 0.64685277263316520724)

001 (start 1.01715544349068021113, decay 0.03414807753972484705, inflow 0.14841098069616112665)

111 (start 0.78429632853633235445, decay 1.15687167512167077277)

1 (start 0.42612651925540023790, decay 1.27909690302355838476)

11 (start 0.20031408998020588941, decay 3.82678210586654943270)

01 (start 1.46509204407481430366, decay 0.39535312579006176570)

0001 (start 0.73905370179378993001, decay 3.07978963926015980590)

0 (start 0.40323682120683845831, decay 1.61004865358691229282)

== INPUTS / OUTPUTS ==

Input(s)

control 01

stimulus 011

Output(s) 11

**CHAMPION NETWORK – 2-BIT TASK**

Fitness -0.125170 (non-pruned)

== REACTIONS ==

001 + 001 <-> 000 + 011 [ k-> = 37.068289, k<- = 0.000300 ]

0 + 00 <-> 000 [ k-> = 59.069158, k<- = 0.070370 ]

0001 + 0 <-> 01 + 000 [ k-> = 38.311504, k<- = 0.590721 ]

01 + 011 <-> 111 + 00 [ k-> = 27.949994, k<- = 17.932196 ]

00 + 001 <-> 01 + 000 [ k-> = 36.339652, k<- = 27.400650 ]

001 + 0 <-> 0001 [ k-> = 35.781877, k<- = 0.011584 ]

0111 + 001 <-> 011 + 0011 [ k-> = 20.827080, k<- = 6.018281 ]

1 + 011 <-> 0111 [ k-> = 22.921276, k<- = 0.031690 ]

0 + 111 <-> 0111 [ k-> = 8.251960, k<- = 0.000090 ]

== SPECIES ==

001 (start 0.132249, decay 0.718661)

000 (start 0.739310, decay 0.021344)

111 (start 4.406750, decay 2.445215)

011 (start 0.354732, decay 1.178916)

0001 (start 0.119382, decay 0.958550)

01 (start 1.490495, decay 7.083166)

1 (start 3.339679, decay 1.912192)

00 (start 0.081220, decay 2.797562)

0011 (start 1.077470, decay 5.061476)

0 (start 0.714631, decay 3.215444)

0111 (start 1.135455, decay 1.607334)

== INPUTS / OUTPUTS ==

Input(s)

control 0

stimulus 01

Output(s) 00

**CHAMPION NETWORK – All TASKS SIMULTANEOUSLY**

Fitness -0.244452 (non-pruned)

== REACTIONS ==

001 + 0 <-> 0001 [ k-> = 12.698808, k<- = 0.109468 ]

111 + 011 <-> 11 + 0111 [ k-> = 26.426352, k<- = 0.998036 ]

1 + 1 <-> 11 [ k-> = 6.970691, k<- = 1.349833 ]

011 + 001 <-> 01 + 0011 [ k-> = 0.573617, k<- = 0.196549 ]

1 + 01 <-> 011 [ k-> = 35.462735, k<- = 8.625780 ]

1 + 1 <-> 11 [ k-> = 15.796971, k<- = 3.058989 ]

0 + 111 <-> 11 + 01 [ k-> = 0.615339, k<- = 0.007711 ]

1 + 1 <-> 11 [ k-> = 13.464200, k<- = 2.607262 ]

00 + 01 <-> 0001 [ k-> = 6.745320, k<- = 0.003152 ]

1 + 1 <-> 11 [ k-> = 34.614301, k<- = 6.702852 ]

0 + 0 <-> 00 [ k-> = 16.153675, k<- = 0.018653 ]

11 + 001 <-> 1 + 0011 [ k-> = 9.757152, k<- = 4.199458 ]

0011 + 00 <-> 001 + 001 [ k-> = 1.094965, k<- = 0.019774 ]

0 + 00 <-> 000 [ k-> = 39.461615, k<- = 0.006145 ]

111 + 0111 <-> 011 + 1111 [ k-> = 28.699882, k<- = 2.939847 ]

1 + 01 <-> 011 [ k-> = 10.194819, k<- = 2.479737 ]

011 + 0111 <-> 001 + 1111 [ k-> = 24.971749, k<- = 0.041816 ]

01 + 00 <-> 0001 [ k-> = 8.514831, k<- = 0.003978 ]

0001 + 0 <-> 00 + 001 [ k-> = 3.368486, k<- = 0.451213 ]

1 + 1 <-> 11 [ k-> = 1.702728, k<- = 0.329723 ]

== SPECIES ==

011 (start 0.084794, decay 2.352670)

001 (start 0.299051, decay 3.588118)

111 (start 1.243316, decay 1.324390)

0 (start 2.465287, decay 0.689242)

01 (start 0.802737, decay 5.702237)

0001 (start 2.765299, decay 1.692862)

11 (start 0.080503, decay 0.000762)

0111 (start 0.575217, decay 2.530677)

1 (start 1.091587, decay 0.089327)

0011 (start 1.642108, decay 1.731765, inflow 0.024917)

00 (start 1.332982, decay 2.369254)

000 (start 0.126751, decay 2.249976)

1111 (start 2.159791, decay 7.217516)

== INPUTS / OUTPUTS ==

Input(s)

control 111

stimulus 01

Output(s) 011

***Part 4. Bayesian analysis***

We compare the dynamics of an evolved network’s best “belief” (the output of the regression model) over time for a particular lifetime to the ideal rational belief (the posterior probability). Figures S.4.1 and S.4.2 show these dynamics for several different networks, including networks that were evolved on a different task to the one they are being tested on.


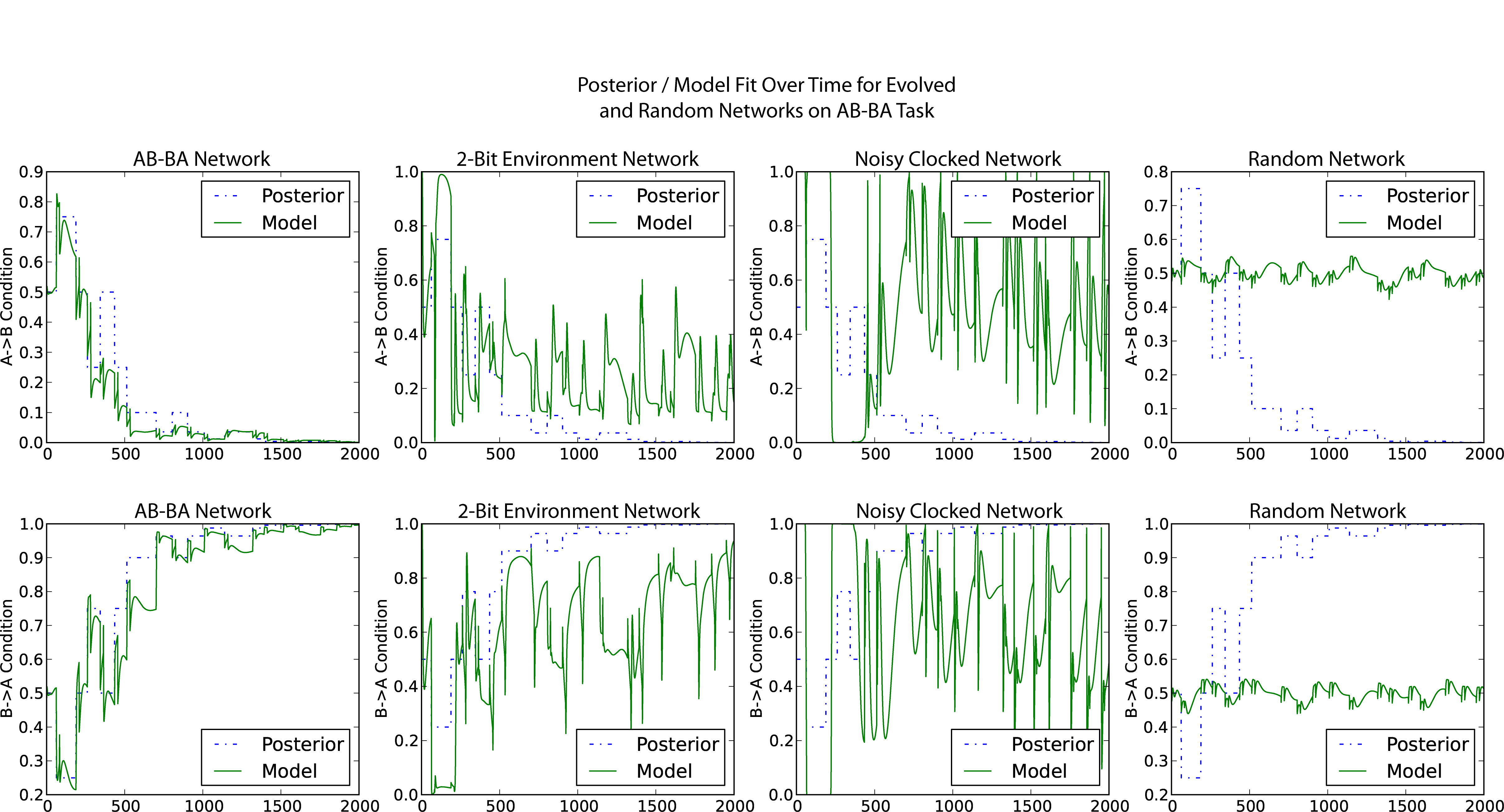


Figure S.4.1. Best “belief” over time for several different evolved networks on two paired runs of the AB-BA task. Upper: Posteriors and fits for a single run in the C→S condition. Lower: Posteriors and fits for a single run in the S→C condition. Left to right: results for an AB-BA-evolved network, a 2-bit-environment-evolved network, a noisy-clocked-evolved network and a random network. Models are fitted to a random sample of AB-BA environments.


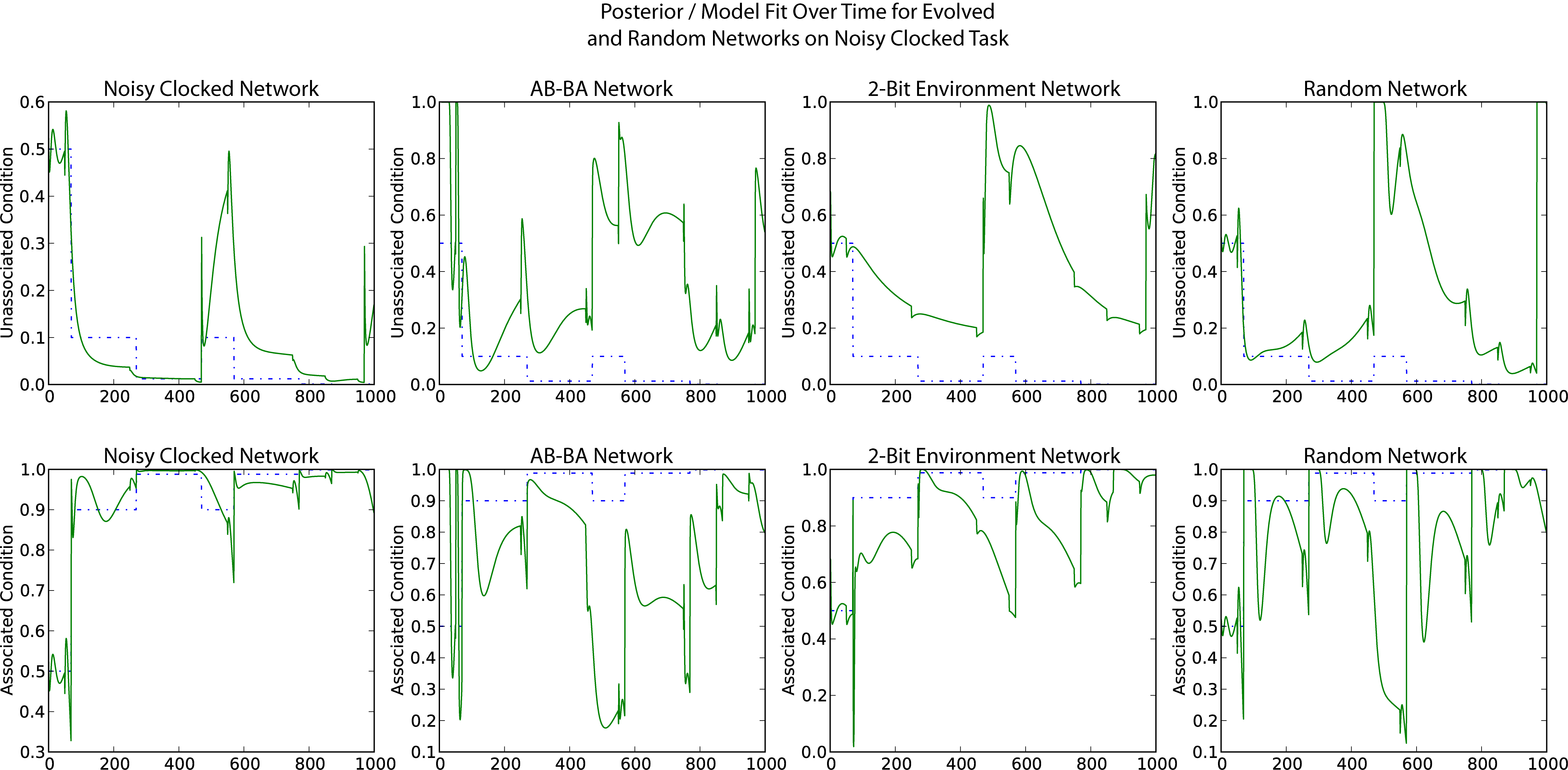


Figure S.4.2. Best “belief” change over time for several different evolved networks on two paired lifetime runs of the noisy clocked task. Upper: Posteriors and fits for a single run in the C→S condition. Lower: Posteriors and fits for a single run in the S→C condition. Left to right: results for an noisy-clocked-evolved network, an AB-BA-evolved network, a 2-bit-environment-evolved network and a random network. Models are fitted to a random sample of noisy clocked environments.

***Part 5. Dependence of Performance on the Rate of Presentation of Stimulus/Control Pairs***


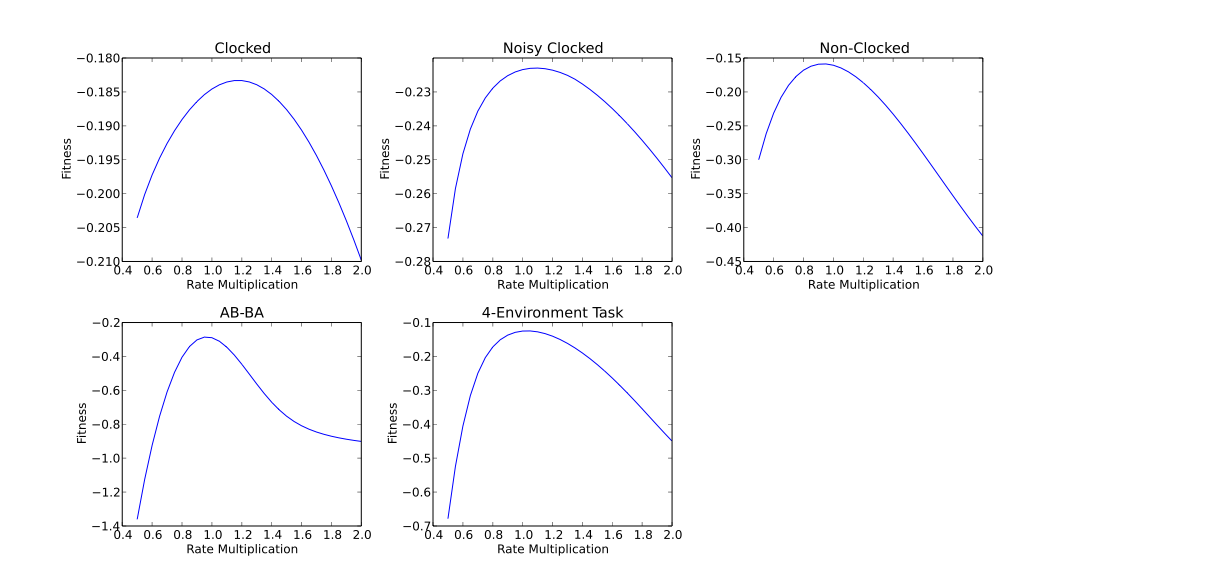


Figure S.5.1: When the rate of presentation of stimuli was increased or decreased compared to the rate of presentation during the training/evolution period, the fitness of the evolved networks dropped off. Thus, associative learning is not maximally general, but rate dependent. The exact nature of the rate dependence varies between evolved networks.

***Part 6. Factors with effects on evolved network fitness***

We evaluated the effects of a variety of different evolutionary conditions on network evolvability. The variants specific to reaction network evolution were:

- Different reaction chemistries (polymer, rearrangement or aggregation)
- Different formula limitations for the chemistries
  - Maximum formula length
  - Number of different "atoms"

and we also investigated a number of standard evolutionary parameters:

- Different spatial embeddings for the population (1d, 2d or non-spatial)
- Different mutation rates
- Different population sizes

Apart from mutation rate, the variables which had the largest effect on fitness after 5000 evaluations were those which constrained the variety of chemical species available to the network: low maximum formula length, minimal chemical alphabet, and a simple aggregation chemistry (Figures S.6.1-4). The results provided are for the 2-bit environment task, which was a relatively non-trivial problem requiring long-term memory, but experiments on other tasks showed a similar pattern.

We investigated the hypothesis that a mutational bias towards more densely connected networks was responsible for the increased fitness seen in networks with smaller chemical alphabets, shorter formula strings and simple aggregate chemistry. Given an evolutionary run, structural mutations which increased the connection density of the network were slightly more likely (9.7%) to be beneficial, compared to structural mutations which decreased the connection density of the network (of which only 8.5% were beneficial). This interaction was significant at the level according to a Yates-corrected test, for each run individually and all taken together. Thus, although mutations which increased the interconnectivity of the network were more likely to be beneficial than mutations which decreased the interconnectivity, we found that there is no straightforward mutation bias toward more densely connected networks in the more successful genetic encodings. Some other factor must therefore account for their improved fitness. Counterintuitively, the frequency of these density-increasing structural mutations was not decreased by using a larger chemical alphabet, longer chemical formulas or a different chemistry. Instead we suspect that constraining the search space into a more readily explorable size might have contributed to the success of simpler networks.


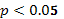

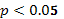

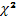

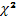

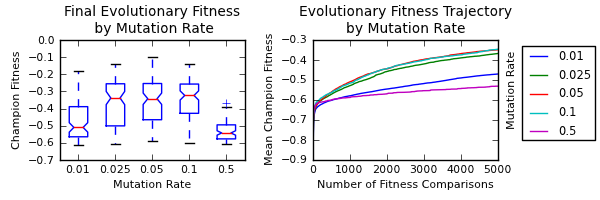


Figure S.6.1. Fitness on the 2-bit environment task as a function of mutation rate. Left: fitness boxplots of champion individuals after 5000 evaluations (N=100), for various mutation rates; right: mean champion fitness by number of evaluations, for various mutation rates.


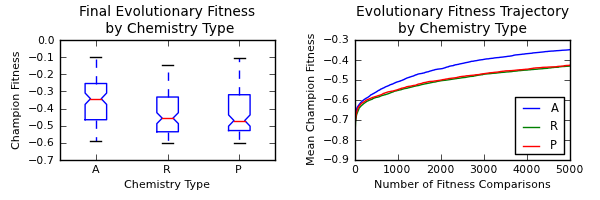


Figure S.6.2. Fitness on the 2-bit environment task as a function of chemistry type. Left: fitness boxplots of champion individuals after 5000 evaluations (N=100), for various chemistry types (A=aggregate, R=rearrangement, P=polymer); right: mean champion fitness by number of evaluations, for various chemistry types.


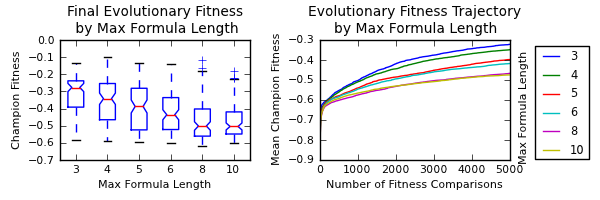


Figure S.6.3. Fitness on the 2-bit environment task as a function of maximum formula length. Left: fitness boxplots of champion individuals after 5000 evaluations (N=100), for various maximum formula lengths; right: mean champion fitness by number of evaluations, for various maximum formula lengths.


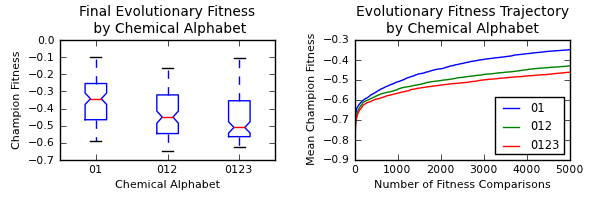


Figure S.6.4. Fitness on the 2-bit environment task as a function of chemical alphabet. Left: fitness boxplots of champion individuals after 5000 evaluations (N=100), for various chemical alphabets; right: mean champion fitness by number of evaluations, for various chemical alphabets.

| **Experiment** | **Higher Connectivity** | | **Lower Connectivity** | |
| --- | --- | --- | --- | --- |
|  | **Deleterious** | **Beneficial** | **Deleterious** | **Beneficial** |
| Benchmark | 10387 (91.8%) | 926 (8.2%) | 6349 (90.5%) | 667 (9.5%) |
| Different Chemistry (Rearrangement vs. Aggregate) | 10810 (91.4%) | 1021 (8.6%) | 7546 (90.5%) | 796 (9.5%) |
| Larger Alphabet  (4 symbols vs. 2) | 11106 (91.3%) | 1054 (8.7%) | 6371 (90.0%) | 708 (10.0%) |
| Longer Formulas  (6 max. length vs. 4) | 10328 (91.5%) | 959 (8.5%) | 7090 (90.3%) | 760 (9.7%) |
| Overall | 42631 (91.5%) | 3960 (8.5%) | 27356 (90.3%) | 2931 (9.7%) |

Table S.6.1. Total number of structural mutations by fitness effect and connectivity effect. Statistics are collated for 10 runs of each experimental condition.

Figures S.6.5. and S.6.6 show effects of population spatial organisation and population size on champion fitness after 5000 fitness comparisons. The effects are small compared to the effects of mutation rate and network encoding.


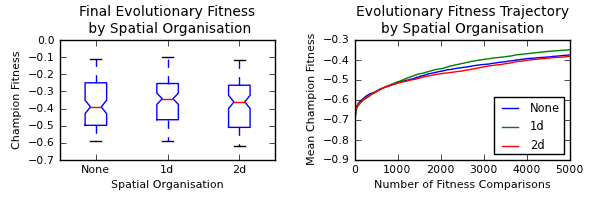


Figure S.6.5: Fitness on the 2-bit environment task as a function of population spatial organisation. Left: fitness boxplots of champion individuals after 5000 evaluations (N=100), for various population types; right: mean champion fitness by number of evaluations, for various population types.


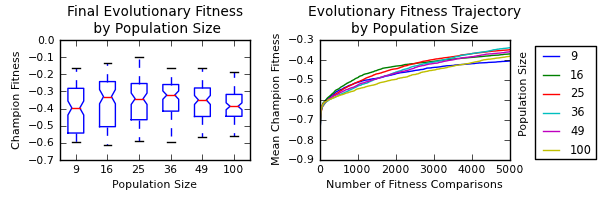


Figure S.6.6: Fitness on the 2-bit environment task as a function of population size. Left: fitness boxplots of champion individuals after 5000 evaluations (N=100), for various population sizes; right: mean champion fitness by number of evaluations, for various population sizes.
